# High-Performance Wide-Temperature Zinc-Ion Batteries with K^+^/C_3_N_4_ Co-Intercalated Ammonium Vanadate Cathodes

**DOI:** 10.1007/s40820-025-01892-0

**Published:** 2025-09-01

**Authors:** Daming Chen, Jimin Fu, Yang Ming, Wei Cai, Yidi Wang, Xin Hu, Rujun Yu, Ming Yang, Yixin Hu, Benjamin Tawiah, Shuo Shi, Hanbai Wu, Zijian Li, Bin Fei

**Affiliations:** 1 Materials Synthesis and Processing Lab, School of Fashion and Textiles, The Hong Kong Polytechnic University, Kowloon, 999077 Hong Kong SAR People’s Republic of China; 2 Department of Applied Biology and Chemical Technology, The Hong Kong Polytechnic University, Kowloon, 999077 Hong Kong SAR People’s Republic of China; 3 College of Chemistry and Environmental Engineering, Shenzhen University, Shenzhen, 518060 People’s Republic of China

**Keywords:** K^+^ and C_3_N_4_ co-intercalation, Synergistic effect, Reaction kinetics, Extreme environments, Aqueous zinc-ion batteries

## Abstract

NH_4_V_4_O_10_ (NVO) is considered a promising cathode material for aqueous zinc-ion batteries due to its high theoretical capacity. However, its practical application is limited by irreversible deamination, structural collapse, and sluggish reaction kinetics during cycling. Herein, K^+^ and C_3_N_4_ co-intercalated NVO (KNVO-C_3_N_4_) nanosheets with expanded interlayer spacing are synthesized for the first time to achieve high-rate, stable, and wide-temperature cathodes. Molecular dynamics and experimental results confirm that there is an optimal C_3_N_4_ content to achieve higher reaction kinetics. The synergistic effect of K^+^ and C_3_N_4_ co-intercalation significantly reduces the electrostatic interaction between Zn^2+^ and the [VO_n_] layer, improves the specific capacity and cycling stability. Consequently, the KNVO-C_3_N_4_ electrode displays outstanding electrochemical performance at room temperature and under extreme environments. It exhibits excellent rate performance (228.4 mAh g^−1^ at 20 A g^−1^), long-term cycling stability (174.2 mAh g^−1^ after 10,000 cycles at 20 A g^−1^), and power/energy density (210.0 Wh kg^−1^ at 14,200 W kg^−1^) at room temperature. Notably, it shows remarkable storage performance at − 20 °C (111.3 mAh g^−1^ at 20 A g^−1^) and 60 °C (208.6 mAh g^−1^ at 20 A g^−1^). This strategy offers a novel approach to developing high-performance cathodes capable of operating under extreme temperatures.

**Supplementary Information:**

The online version contains supplementary material available at 10.1007/s40820-025-01892-0.

## Highlights

- Molecular dynamics and experimental results confirm that adjusting the interlayer spacing by changing the C_3_N_4_ content effectively improves the reaction kinetics.
- The synergistic effect of K^+^ and C_3_N_4_ co-intercalation lowers the energy barrier, reduces the electrostatic interaction, and enhances the kinetics and structural stability.
- The K^+^/C_3_N_4_ co-intercalated NH_4_V_4_O_10_ cathode exhibits excellent electrochemical performance at room temperature and under extreme environments.

**Supplementary Information:**

The online version contains supplementary material available at 10.1007/s40820-025-01892-0.

## Graphical abstract

**Figure:**
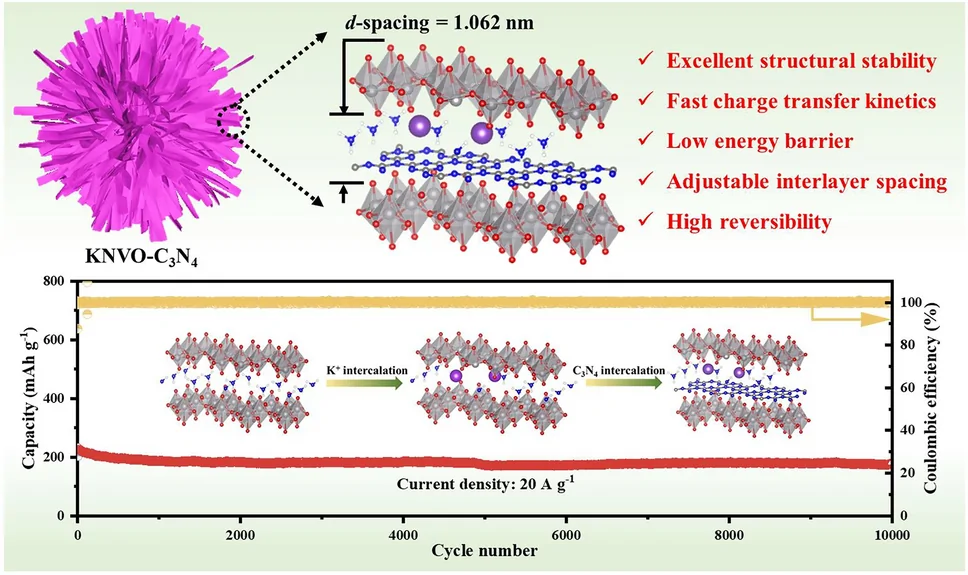

## Introduction

Aqueous zinc-ion batteries (AZIBs) have garnered significant interest due to their high safety, low cost, and environmental friendliness, positioning them as promising candidates for the next generation of efficient rechargeable batteries [1–4]. Although the zinc anode possesses a high theoretical specific capacity (820 mAh g^−1^) and a relatively low electrochemical potential (−0.76 V vs. standard hydrogen electrode) [5–7]. However, the absence of a matching cathode with high capacity, satisfactory cycling stability, and enhanced ion diffusion has severely impeded its practical application [8, 9]. Consequently, the development of an appropriate cathode remains a critical and meaningful challenge.

Recent advancements in cathode material research have made remarkable progress, mainly including vanadium (V)-based oxides, manganese-based oxides, Prussian blue analogs, and conductive metal–organic framework [10–14]. V-based oxides have emerged as a focal point of the current research due to their high theoretical specific capacity, multi-electron transfer capability of V elements, and abundant reserves [15]. Among the various V-based oxides, layered ammonium vanadate (NH_4_V_4_O_10_, NVO) is regarded as a promising candidate owing to several advantages [16, 17]: (i) Its larger interlayer spacing (9.8 Å) facilitates the insertion/extraction of hydrated Zn^2+^. (ii) It offers higher specific capacity, thereby providing enhanced energy density and power density. (iii) NH^4+^ forms N–H···O hydrogen bonds with the [VO_n_] layer, improving the structural stability and electrochemical performance of the cathode electrode. Nonetheless, research indicates that irreversible deammoniation occurs during the charge/discharge process, resulting in significant phase transitions and structural degradation. Additionally, the excess NH^4+^ between the V–O layers may seriously impede the insertion/extraction of Zn^2+^ due to strong electrostatic interactions [18].

To address the aforementioned challenges and improve the electrochemical performance of NVO cathodes, various strategies such as defect engineering, doping, and surface coating have been developed. A particularly effective approach involves substituting part of NH^4+^ with cations (e.g., Na^+^, K^+^, Rb^+^, Zn^2+^, Mg^2+^, and Ti^4+^), which effectively suppresses deammoniation during the charge/discharge process and improves the structural stability, conductivity, and the specific capacity. [16, 17, 19–22] Additionally, studies have found that the introduction of multivalent cations with larger radius (e.g., Zn^2+^, Mg^2+^, and Mn^2+^) can increase the interlayer spacing of the host material, thereby accelerating ion diffusion [20, 23]. However, the electrochemical stability of the host material requires further enhancement, as foreign cations may also de-intercalate during the charge/discharge process, leading to structural collapse. As for monovalent cations, theoretical calculations suggest that compared to Li^+^ and Na^+^, the introduction of K^+^ into V-based oxides to form K–O bonds can more effectively improve the crystal structure and enhance the reaction kinetics [24]. For instance, Zong et al. demonstrated that K^+^ pre-intercalated NVO significantly improved diffusion kinetics and alleviated irreversible deammoniation, resulting in excellent electrochemical performance [16]. Nonetheless, the electrostatic interaction between Zn^2+^ and the V–O layer still needs to be further weakened to lower the de-solvation energy barrier and enhance the reaction kinetics.

Pre-intercalating two guest species, such as cations and organic materials, into the interlayer of host material represents a significant and effective strategy [25]. For instance, Zhao et al. have demonstrated that the simultaneous introduction of Na^+^ and polyaniline into NVO can increase the interlayer spacing, thereby reducing the Zn^2+^ diffusion barrier and enhancing structural stability [26]. Consequently, to facilitate rapid Zn^2+^ diffusion, mitigate electrostatic interactions, and preserve structural integrity, it is urgent to co-intercalate cations and organic materials in NVO to optimize the electrochemical performance. Nonetheless, research on the simultaneous co-intercalation of cations and organic materials in NVO remains limited. Notably, systematic research reports on the adjustment of NVO interlayer spacing by organic materials to enhance zinc ion storage are also scarce.

Recent advancements have revealed that incorporating graphite-phase carbon nitride (g-C_3_N_4_) into the host material enhances structural and chemical stability during the cycling process [27]. Herein, we prepared a novel K^+^ and C_3_N_4_ co-intercalated NVO (KNVO-C_3_N_4_) nanosheet electrode with its interlayer spacing adjustable by varying the C_3_N_4_ content. It is found that the intercalation of K^+^ more effectively enhances the specific capacity of NVO, whereas the intercalation of C_3_N_4_ is more beneficial for improving the stability of NVO. The synergistic effect of K^+^ and optimal C_3_N_4_ co-intercalation effectively reduces the electrostatic interaction between Zn^2+^ and the [VO_n_] layer, lowers the adsorption energy barrier, and enhances the reaction kinetics, which is confirmed by experimental data and density functional theory (DFT) calculations. These advantages endow the KNVO-C_3_N_4_ electrode with excellent rate performance, long-term cycling stability, and energy/power density at room temperature electrochemical tests, surpassing most reported V-based oxide cathode materials. Additionally, the constructed pouch cell displays outstanding cycling stability at various bending angles and exhibits remarkable storage performance under extreme temperature environments, indicating promising application potential.

## 2 Experimental

### Synthesis of K^+^ Intercalated NH_4_V_4_O_10_ (KNVO)

NH_4_VO_3_ (4 mmol), H_2_C_2_O_4_·2H_2_O (4.8 mmol), and KCl (0.4 mmol) were dissolved in 60 mL of deionized water and stirred at 80 °C for 30 min. The resulting solution was transferred into a 100-mL autoclave equipped with a Teflon liner and maintained at 180 °C for 4 h. Subsequently, the mixture was washed several times with deionized water and ethanol, followed by drying in a vacuum oven at 80 °C for 12 h to yield K^+^ intercalated NH_4_V_4_O_10_ (referred to as KNVO). When KCl was omitted from the procedure, while all other experimental conditions were kept constant, the resulting product was NH_4_V_4_O_10_ (referred to as NVO).

### Synthesis of K^+^ and C_3_N_4_ Co-intercalated NH_4_V_4_O_10_ (KNVO/C_3_N_4_)

As reported previously [28], urea was calcined at 550 °C in a tubular furnace for 2 h to synthesize C_3_N_4_, under an air atmosphere with a heating rate of 5 °C min^−1^. Subsequently, 15 mg of C_3_N_4_ and 300 mg of KNVO were dispersed in 100 mL of deionized water and stirred at 80 °C for 5 h. The mixture was then washed several times with deionized water and ethanol, followed by drying in a vacuum oven at 80 °C for 12 h, resulting in K^+^ and C_3_N_4_ co-intercalated NH_4_V_4_O_10_ (referred to as KNVO-C_3_N_4_). Similarly, when the C_3_N_4_ content was adjusted to 7.5 mg and 30 mg while maintaining other reaction conditions, the resulting samples were referred to as KNVO-C_3_N_4_-2.5% and KNVO-C_3_N_4_-10%, respectively. When NVO was used instead of KNVO, C_3_N_4_ intercalated NH_4_V_4_O_10_ can be obtained (referred to as NVO-C_3_N_4_). When the KCl content was adjusted to 0.2 and 0.6 mmol while maintaining other reaction conditions, the resulting samples were referred to as KNVO-C_3_N_4_-1 and KNVO-C_3_N_4_-2, respectively.

### Characterization

Scanning electron microscopy (SEM, Tescan MIRA) and transmission electron microscopy (TEM, FEI Tecnai F20) were used to observe the morphology and structure of the samples. The crystal structures of the samples were measured by X-ray diffraction (XRD, Rigaku SmartLab 9 kW-Advance). The molecular and chemical structure were measured by Fourier transform infrared spectroscopy (FTIR, Nicolet iS 10) and Raman spectroscopy (Thermo Fisher). X-ray photoelectron spectroscopy (XPS, Thermo Scientific ESCALAB 250Xi) was used to detect the electronic states and surface elements. The oxygen vacancy was performed through the electron paramagnetic resonance (EPR, Bruker EMXplus-6/1) experiments.

### Electrochemical Measurements

Battery Assembly: For the evaluation of coin-type cells, the working electrodes were prepared by mixing the active materials (NVO, KNVO, NVO-C_3_N_4_, KNVO-C_3_N_4_-2.5%, KNVO-C_3_N_4_, KNVO-C_3_N_4_-10%, KNVO-C_3_N_4_-1, KNVO-C_3_N_4_-2, and C_3_N_4_), acetylene black, and polyvinylidene fluoride (PVDF) in N-methyl-2-pyrrolidone (NMP) at a mass ratio of 7:2:1 to form a uniform slurry. This slurry was uniformly coated onto stainless-steel mesh and dried at 90 °C overnight under vacuum conditions. The electrodes were subsequently punched into circular disks with a diameter of 12 mm. The mass of the slurry was determined by subtracting the mass of the bare current collector from that of the dried electrode. Based on the mass ratio of the slurry, the mass of active material was calculated to be approximately 1.8 to 2 mg per disk. The CR2032-type coin cells were assembled using a zinc foil anode, a glass fiber (Whatman GF/F) separator, and a 3 M aqueous Zn(CF_3_SO_3_)_2_ electrolyte. In the pouch cell assembly, zinc foil and KNVO-C_3_N_4_ (with a mass loading of about 15 mg) served as the anode and cathode, and the electrolyte is PAM gel with good low-temperature tolerance [29].

Electrochemical Testing: Galvanostatic charge/discharge (GCD) measurements were conducted by using a LAND CT3001A battery testing system (Wuhan, China) within a voltage range of 0.2–1.6 V. Cyclic voltammetry (CV) was conducted with scan rates ranging from 0.2 to 1.0 mV s⁻^1^, and electrochemical impedance spectroscopy (EIS) was carried out over a frequency range of 0.1 to 10^6^ Hz using a CHI-760E electrochemical workstation (Shanghai Chenhua). The galvanostatic intermittent titration technique (GITT) was employed during the second cycle to determine the Zn^2^⁺ diffusion coefficient, employing a current pulse of 0.5 A g^−1^ for 20 min, followed by a relaxation period of 30 min.

## Results and Discussion

### Microstructure and Composition Analysis

**Fig. 1:**
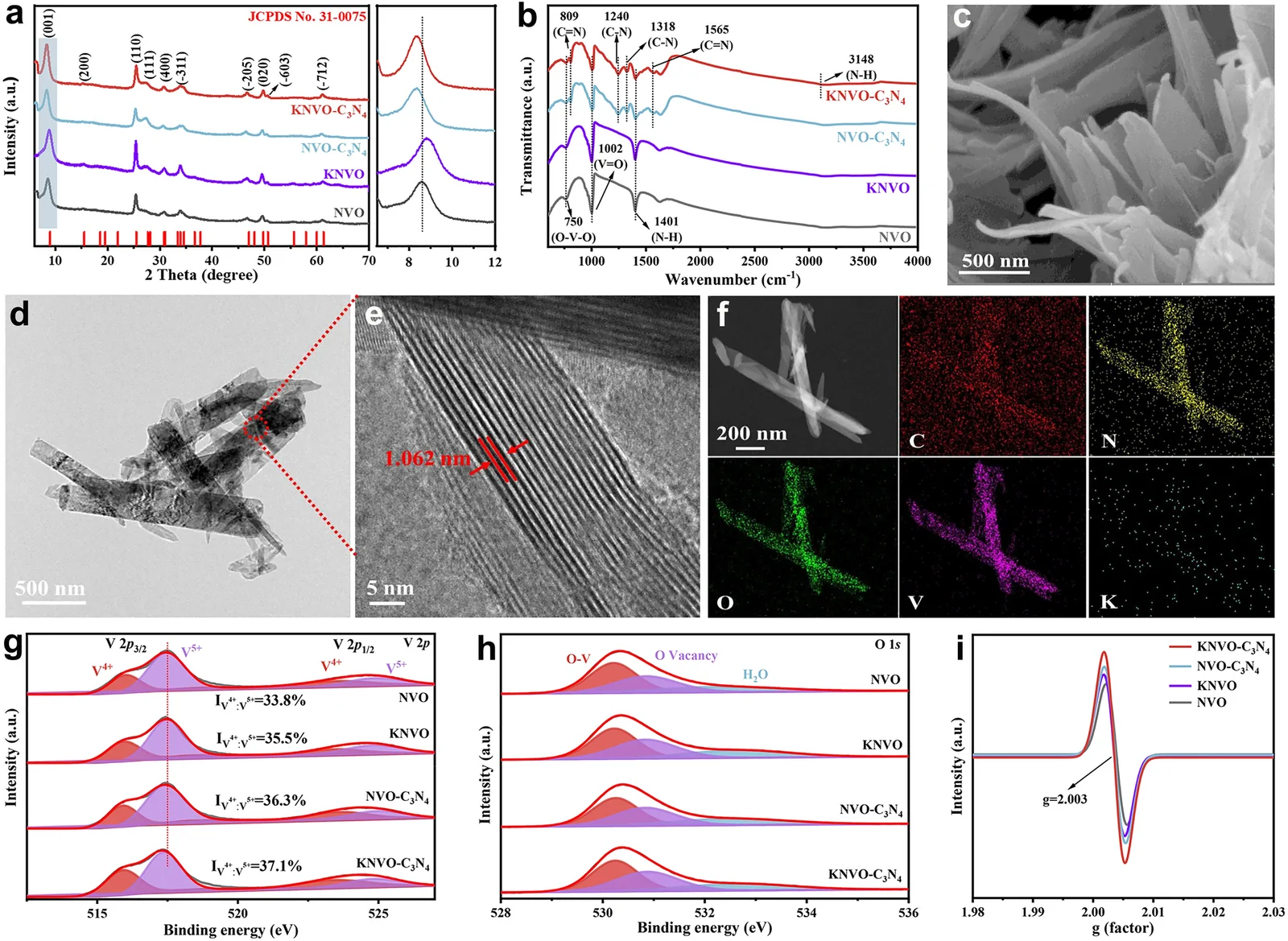
**a** XRD pattern of NVO, KNVO, NVO-C_3_N_4_, and KNVO-C_3_N_4_, respectively. **b** FTIR spectra of NVO, KNVO, NVO-C_3_N_4_, and KNVO-C_3_N_4_, respectively. **c-e** SEM, TEM, and HRTEM images of KNVO-C_3_N_4_. **f** HAADF-STEM image of KNVO-C_3_N_4_ and the elemental distribution of C, N, O, V and K. **g**,** h** XPS spectra of V 2*p* and O 1*s.* **i** EPR spectra of NVO, KNVO, NVO-C_3_N_4_, and KNVO-C_3_N_4_, respectively

K^+^ and C_3_N_4_ co-intercalated NH_4_V_4_O_10_ (KNVO-C_3_N_4_) was synthesized using straightforward hydrothermal and low-temperature stirring methods, as depicted in Fig. S1. Control samples, including NH_4_V_4_O_10_ (NVO), K^+^ intercalated NH_4_V_4_O_10_ (KNVO), C_3_N_4_ intercalated NH_4_V_4_O_10_ (NVO-C_3_N_4_), and K^+^ and various C_3_N_4_ contents co-intercalated NH_4_V_4_O_10_ (KNVO-C_3_N_4_-2.5% or KNVO-C_3_N_4_-10%) were also prepared. Detailed procedures are provided in the experimental section. The crystal structures of the samples (NVO, KNVO, NVO-C_3_N_4_, and KNVO-C_3_N_4_) were characterized by XRD. Figure 1a shows the above-prepared samples are well indexed to the pure monoclinic NH_4_V_4_O_10_ phase with a space of *C*2/m (JCPDS No. 31-0075) [30]. Following the introduction of K^+^ and C_3_N_4_ (Fig. S2), no additional diffraction peaks are detected. Notably, the (001) phase of KNVO shifts rightward compared to NVO, attributed to the partial substitution of NH_4_^+^ by K^+^ [16]. Conversely, the (001) phase of NVO-C_3_N_4_ and KNVO-C_3_N_4_ shifts leftward, indicating that the intercalation of C_3_N_4_ into NVO and KNVO can increase the interlayer spacing. The interlayer spacing of KNVO-C_3_N_4_ expands to 10.62 Å compared to 9.96 Å for NVO. In addition, the extent of the leftward shift of the (001) phase (Fig. S3) increases with higher C_3_N_4_ content, demonstrating that greater C_3_N_4_ content facilitates larger interlayer spacing.

The FTIR of NVO, KNVO, C_3_N_4_, NVO-C_3_N_4_, and KNVO-C_3_N_4_ is presented in Figs. 1b and S4. The characteristic vibration peaks at 750 and 1002 cm^−1^ correspond to O–V–O and V=O [16, 17]. Peaks at 809 and 1565 cm^−1^ correspond to C=N, and those at 1240 and 1318 cm^−1^ correspond to C–N [31, 32]. It is evident that NVO-C_3_N_4_ and KNVO-C_3_N_4_ exhibit characteristic peaks of both C_3_N_4_ and NH_4_V_4_O_10_, confirming the presence of C_3_N_4_ within NVO [31]. Raman spectra (Fig. S5) reveal six distinct vibration peaks at 145, 195, 286, 410, 698, and 995 cm^−1^, originating from the bending and stretching models of the –V–O–V–O– chain and V = O bond in the VO_6_ octahedron [20, 29].

The morphological characteristics of the synthesized samples were investigated using SEM and TEM. As depicted in Fig. S6, KNVO-C_3_N_4_ exhibits a 3D flower-like morphology composed of numerous nanosheets. In comparison to NVO (Figs. 1c, S7, and S8), the intercalation of K^+^ and C_3_N_4_ has not destroyed the morphology and structure of the material. TEM and HRTEM images (Figs. 1d, e and S9) of KNVO-C_3_N_4_ and NVO show lattice fringes of 1.062 and 0.996 nm, respectively, corresponding to the (001) plane of the materials, which is consistent with the XRD findings. Furthermore, the high-angle annular dark field scanning TEM (HAADF-STEM) image (Fig. 1f) confirms the uniform distribution of C, N, O, V, and K elements in KNVO-C_3_N_4_, indicating that the successful introduction of K^+^ and C_3_N_4_. Additionally, the Brunauer–Emmett–Teller (BET) analysis (Fig. S10) indicates that the samples possess a low specific surface area, which is advantageous for minimizing side reactions [33].

The surface chemical state and elemental composition of the synthesized samples were further examined using XPS. The XPS spectra presented in Fig. S11 reveal detectable signal peaks for N, O, and V across all samples. Notably, the K peak is observed exclusively in the KNVO and KNVO-C_3_N_4_ samples, once again confirming the successful introduction of K^+^. The high-resolution K 2*p* spectrum displays two peaks, K 2*p*_*1/2*_ at 295.0 eV and K 2*p*_*3/2*_ at 292.1 eV, as illustrated in Fig. S12 [34]. Compared to the NVO and KNVO samples, the N peak intensity in NVO-C_3_N_4_ and KNVO-C_3_N_4_ is significantly increased, which can be attributed to the successful introduction of C_3_N_4_. The N 1*s* peak (Fig. S13) of NVO, with a binding energy of 400.7 eV, is attributed to NH_4_^+^, whereas the N 1*s* peaks of C_3_N_4_ at 398.3 and 399.8 eV correspond to the *sp*^2^ hybridization of C–N=C and N–(C)_3_ coordination [31, 35]. These three signal peaks are simultaneously detected in KNVO-C_3_N_4_, further proving the successful intercalation of C_3_N_4_ into NVO or KNVO. In addition, the C–N=C of KNVO-C_3_N_4_ exhibits a slight shift relative to that of C_3_N_4_, which may be attributed to an increase in the electron density of C_3_N_4_ caused by the intercalation of C_3_N_4_ into KNVO to form a heterostructure [36].

In the high-resolution V 2*p* spectrum of NVO (Fig. 1g), four signal peaks are observed at 516.0/523.1and 517.5/524.4 eV, responding to the bending energies of V^4+^ and V^5+^ [20, 37]. Compared to NVO, the characteristic peaks of KNVO, NVO-C_3_N_4_, and KNVO-C_3_N_4_ shift toward lower binding energies, likely due to an increase in the electron density of V following the loss of adjacent oxygen atoms caused by the introduction of K^+^ and C_3_N_4_. Based on the fitting peak area, the ratio of V^4+^ in KNVO and NVO-C_3_N_4_ is higher than that in NVO, indicating that part of the V^5+^ may be reduced to V^4+^ after K^+^ or C_3_N_4_ intercalation, which is conducive to the generation of oxygen vacancies (OVs) [26, 30]. Meanwhile, the V^4+^/V^5+^ ratio is highest in KNVO-C_3_N_4_, suggesting that the co-intercalation of K^+^ and C_3_N_4_ generates more OVs. The O 1*s* spectrum (Fig. 1h) reveals three peaks at 530.3, 531.1, and 532.6 eV, belonging to lattice oxygen (O–V bond), OVs, and oxygen in absorbed water, respectively [38, 39]. Compared to NVO (Fig. 1h and Table S1), the peak area ratio of OVs increases in KNVO and NVO-C_3_N_4_, indicating that the intercalation of K^+^ and C_3_N_4_ can effectively enhance the concentration of OVs. Moreover, KNVO-C_3_N_4_ exhibits the largest peak area ratio of OVs, demonstrating that the co-intercalation of K^+^ and C_3_N_4_ elevates the OVs concentration, thereby effectively improving reaction kinetics. The OVs in these samples are further assessed by EPR measurement (Fig. 1i), where KNVO-C_3_N_4_ also shows the highest OVs concentration, confirming that the intercalation of K^+^ or C_3_N_4_ helps to increase the OVs concentration in NVO, consistent with the O 1*s* spectrum results. In addition, the synergistic effect of the co-intercalation of K^+^ and C_3_N_4_ is more conducive to increasing the OVs concentration, further improving the conductivity of the host material.

### Electrochemical Performance

**Fig. 2:**
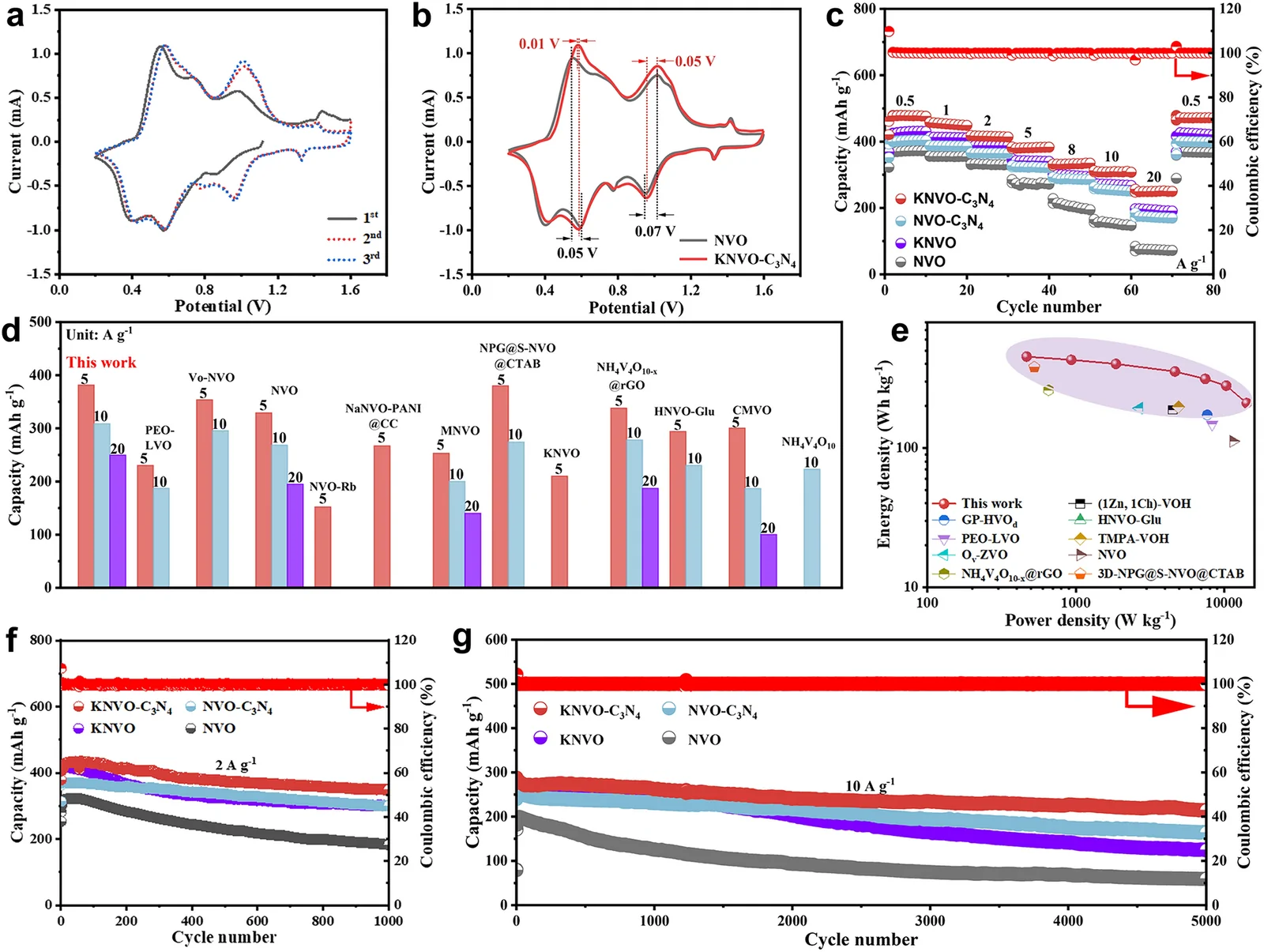
**a** The initial three CV curves of the KNVO-C_3_N_4_ electrode were recorded at 0.2 mV s^−1^. **b** CV curves of NVO and KNVO-C_3_N_4_ after the fifth cycle at 0.2 mV s^−1^. **c** Rate performances of NVO, KNVO, NVO-C_3_N_4_, and KNVO-C_3_N_4_. **d** Rate performance of KNVO-C_3_N_4_ cathode compared with the literatures. **e** Ragone plot of KNVO-C_3_N_4_ cathode compared with literatures. **f**,** g** Cycling performances of NVO, KNVO, NVO-C_3_N_4_, and KNVO-C_3_N_4_ at 2 and 10 A g^−1^, respectively

The electrochemical performances of the samples were further evaluated by assembling CR2032 coin cells using a 3 M Zn(CF_3_SO_3_)_2_ aqueous electrolyte. Figures 2a and S14 present the CV curves of NVO and KNVO-C_3_N_4_ electrodes at a scan rate of 0.2 mV s^−1^ within a voltage range of 0.2–1.6 V. The three redox peaks at 1.03/0.96, 0.73/0.58, and 0.54/0.40 V correspond to the V^5+^/V^4+^ and V^4+^/V^3+^ redox pairs during the Zn^2+^ intercalation/de-intercalation process [15, 20]. A pair of weaker redox peaks at 1.42/1.33 V is related to the Zn^2+^ insertion/extraction in the new phase generated during the cycling process [19]. Figure 2b compares the CV curve of NVO and KNVO-C_3_N_4_ electrodes after the fifth cycle. The KNVO-C_3_N_4_ cathode exhibits a smaller voltage gap on both the V^5+^/V^4+^ and V^4+^/V^3+^ redox pairs, suggesting enhanced ion diffusion and redox reaction kinetics due to the co-intercalation of K^+^ and C_3_N_4_ [15, 40].

Figure 2c and Table S2 illustrate the rate performance of the four electrodes (NVO, KNVO, NVO-C_3_N_4_, and KNVO-C_3_N_4_) as the current density gradually increases from 0.5 to 20 A g^−1^. The KNVO-C_3_N_4_ electrodes exhibit specific capacities of 478.5, 453.2, 415.9, 381.3, 332.1, 308.5, and 249.6 mAh g^−1^, significantly surpassing those of NVO (74.7 mAh g^−1^ at 20 A g^−1^), KNVO (194.2 mAh g^−1^ at 20 A g^−1^), and NVO-C_3_N_4_ (172.6 mAh g^−1^ at 20 A g^−1^) electrodes, demonstrating superior rate performance. Upon returning the current density to 0.5 A g^−1^, the specific capacity recovers to 472.5 mAh g^−1^, indicating excellent rate capability. The KNVO-C_3_N_4_ electrode exhibits a rate retention of 52.2% (0.5/20 A g^−1^), surpassing that of the KNVO electrode (45.2%). This result demonstrates that the intercalation of C₃N₄ effectively enhances the rate performance. Figures S15a and S16a show the rate performance of intercalated samples with varying C_3_N_4_ contents and pure C_3_N_4_ electrodes. The KNVO-C_3_N_4_ electrode consistently exhibits the best rate performance, while the KNVO-C_3_N_4_-10% is not satisfactory. Although the excessive intercalation of poorly conductive C_3_N_4_ increases the interlayer spacing, it hinders the rapid transfer and intercalation of Zn^2+^ at high current densities, leading to diminished rate performance [39]. Additionally, C_3_N_4_ contributes little to specific capacity (Fig. S16b), reducing the content of active substances and hindering specific capacity enhancement. Therefore, an optimal C_3_N_4_ intercalation content can further improve the reaction kinetics and specific capacity of the material. Moreover, the KNVO-C_3_N_4_ electrode (Fig. 2d) demonstrates competitive rate performance (249.6 mAh g^−1^ at 20 A g^−1^) compared to the previously reported V-based cathodes for AZIBs [10, 15–17, 20, 26, 31, 37, 39, 41–43]. Impressively, the prepared KNVO-C_3_N_4_ electrode (Fig. 2e) also shows significant advantages in energy density and power density (452.6 Wh kg^−1^ at 466.6 W kg^−1^ and 210.0 Wh kg^−1^ at 14,200 W kg^−1^) compared to other reported AZIBs cathodes [10, 15, 25, 31, 39, 42, 44–46].

Figure S17 presents the cycling tests of the four electrodes at a low current density of 0.5 A g^−1^. Among them, the KNVO-C_3_N_4_ electrode achieves a notable specific capacity (462.2 mAh g^−1^) and a coulombic efficiency (CE) close to 100% after 50 cycles. Additionally, the long-term cycling performance of the electrode is further evaluated, as shown in Fig. 2f. The KNVO-C_3_N_4_ electrode also displays the highest specific capacity of 348.5 mAh g^−1^ after 1,000 cycles at a current density of 2 A g^−1^. Even at a higher current density of 10 A g^−1^ (Fig. 2g), the specific capacity of the KNVO-C_3_N_4_ electrode after 5000 cycles is 214.2 mAh g^−1^, with a capacity retention rate of 78.2%, which is higher than that of NVO (33.6%), KNVO (46.8%), and NVO-C_3_N_4_ (63.7%). These performance results prove that K^+^ intercalation is more beneficial to increasing the specific capacity of the host material than that of C_3_N_4_, while C_3_N_4_ intercalation contributes more to stability. This is primarily due to the increased interlayer spacing, which effectively enhances the reversibility of Zn^2+^ insertion/extraction and reaction kinetics, thereby alleviating structural collapse. Consequently, the co-intercalation of K^+^ and C_3_N_4_ significantly enhances the cycling stability and specific capacity of the electrodes. In addition, the rate performance and cycling stability of intercalation with different K^+^ contents were compared (Fig. S18). Among them, the KNVO-C_3_N_4_ electrode shows the best electrochemical performance, indicating that there is an optimal K^+^ intercalation content, which is similar to the research results of Zong et al. [16]. Therefore, the following mainly studies the effects of varying C_3_N_4_ intercalation. Figure S15b shows the impact of varying C_3_N_4_ intercalation on capacity retention. After 800 cycles at a current density of 2 A g^−1^, the capacity retention rates of KNVO-C_3_N_4_-2.5%, KNVO-C_3_N_4,_ and KNVO-C_3_N_4_-10% electrodes are 64.5%, 85.1%, and 79.3%, respectively. After 5000 cycles at a high current density of 10 A g^−1^ (Fig. S15c), the KNVO-C_3_N_4_-10% electrode also exhibits lower capacity retention than the KNVO-C_3_N_4_ electrode, suggesting that excessive C_3_N_4_ intercalation may destroy the stability of the structure and adversely affect cycling stability [47]. Therefore, the excellent rate performance and cycling stability of the KNVO-C_3_N_4_ electrode are attributed to the synergistic effect of K^+^ and optimal C_3_N_4_ co-intercalation. The KNVO-C_3_N_4_ electrode retains a specific capacity of 174.2 mAh g^−1^ after 10,000 cycles at a high current density of 20 A g^−1^ (Fig. S19), demonstrating outstanding long-term cycling stability. Additionally, the KNVO-C_3_N_4_ electrode maintains the nanosheet structure after 100 cycles at a current density of 2 A g^−1^, indicating excellent structural stability (Fig. S20). Furthermore, the KNVO-C_3_N_4_ electrode exhibits superior electrochemical performance compared to other V-based oxide cathodes reported in recent literature (Table S3), making it a promising candidate for large-scale energy storage systems.

### Electrochemical Kinetics

**Fig. 3:**
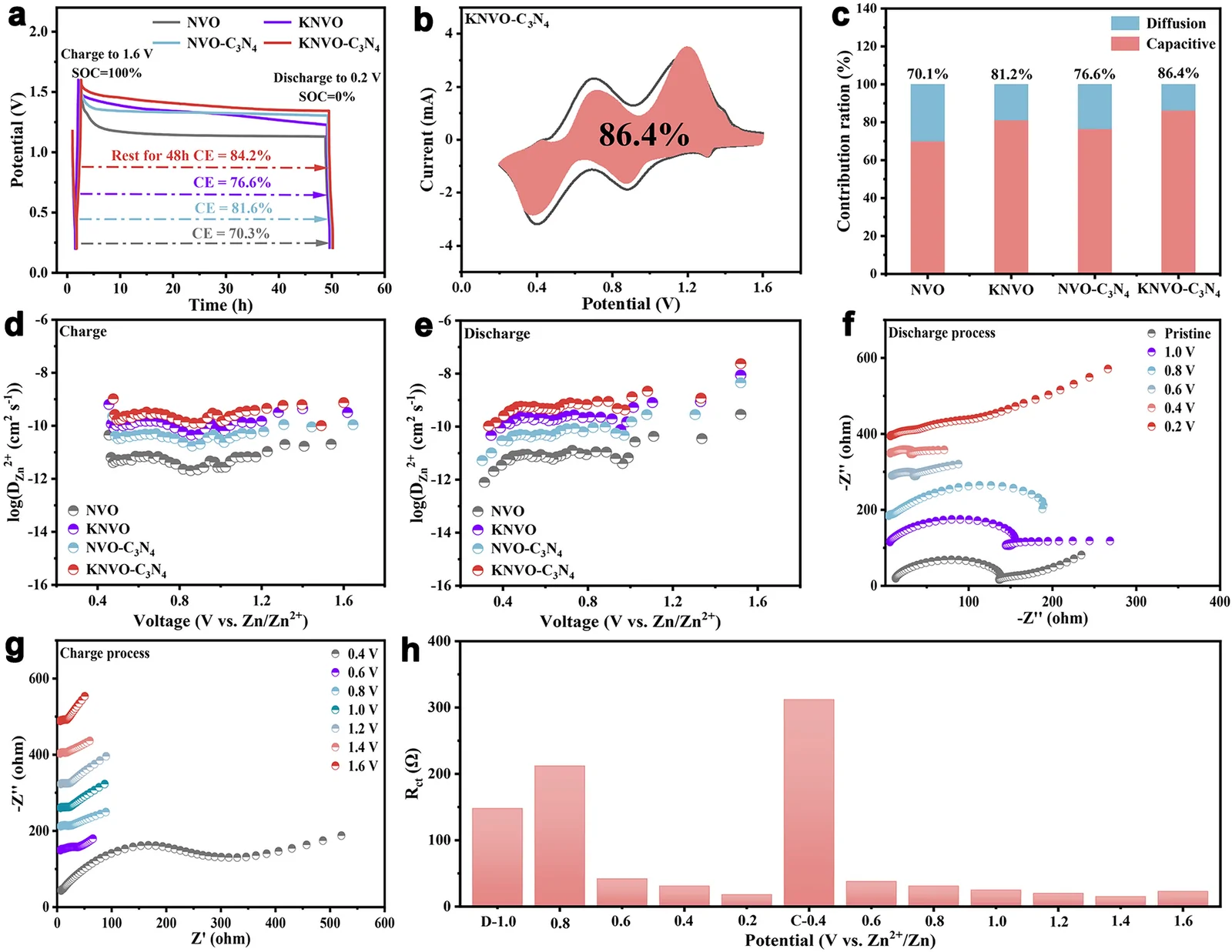
**a** Self-discharge based on the NVO, KNVO, NVO-C_3_N_4_, and KNVO-C_3_N_4_ cathodes. **b** CV curves of KNVO-C_3_N_4_ electrode with capacitive- and diffusion-controlled contributions at 1.0 mV s^−1^. **c** Ratio of capacitive contribution of NVO, KNVO, NVO-C_3_N_4_, and KNVO-C_3_N_4_ electrodes at 1.0 mV s^−1^. **d**, **e** Zn^2+^ diffusion coefficients versus different discharge/charge states. **f**, **g** Nyquist plots for KNVO-C_3_N_4_ electrode during the discharge and charge process. **h** R_ct_ for KNVO-C_3_N_4_ electrode during the discharge and charge process

In the investigation of storage dynamics, the charge retention capacity and stability of the electrode were initially assessed through the self-discharge test (Fig. 3a). After 48 h of rest, the CE of the fully discharged Zn||KNVO-C_3_N_4_ battery is 84.2%, surpassing that of the Zn||NVO (CE = 70.3%), Zn||KNVO (CE = 81.6%), and Zn||NVO-C_3_N_4_ battery (CE = 76.6%), indicating the synergistic effect of K^+^ and C_3_N_4_ co-intercalation in KNVO-C_3_N_4_ contributes to further enhance the stability. The inhibition of self-discharge behavior is mainly due to the co-intercalation of K^+^ and C_3_N_4_, which reinforces the host structure, weakens the interaction between the [VO_n_] layer and Zn^2+^, reduces ion self-diffusion, and thus prevents degradation under open circuit voltage conditions [25, 48].

Subsequently, the electrochemical kinetics of the electrodes were studied using CV curves. Figure S21 displays the CV curves of NVO, KNVO, NVO-C_3_N_4_, and KNVO-C_3_N_4_ electrodes within a scan rate range of 0.2–1.0 mV s^−1^. The b-values of each redox peak can be obtained through the power-law equation ($i=a{v}^{b}$) [49]. Here, a and b are adjustable parameters. When the b value is close to 0.5, the reaction process depends on the ion diffusion process control, and when the b value reaches 1, the corresponding electrochemical behavior is controlled by capacitance. The b values for the KNVO-C_3_N_4_ electrode (Fig. S22a) fall within the range of 0.5–1, proving that its electrochemical reaction is controlled by both capacitance and diffusion control. The pseudo-capacitance contribution ratio of electrodes can be analyzed using the formula ($i\left(V\right)={k}_{1}v+{k}_{2}{v}^{1/2}$), wherein ${k}_{1}$ and ${k}_{2}$ are constants, ${k}_{1}v$ and ${k}_{2}{v}^{1/2}$ represent the contributions of the diffusion and pseudo-capacitance process [50]. At 1.0 mV s^−1^, the pseudo-capacitance contribution rate (Fig. 3b, c) of the KNVO-C_3_N_4_ electrode (86.4%) exceeds that of NVO (70.1%), KNVO (81.2%), and NVO-C_3_N_4_ (76.6%), indicating superior electrochemical kinetics. Furthermore, the high pseudo-capacitance contribution of KNVO-C_3_N_4_ suggests that the K^+^ and C_3_N_4_ co-intercalation may enhance the zinc storage mechanism of NVO. As the scan rate increases, the capacitance contribution of the KNVO-C_3_N_4_ electrode rises from 67.3% to 86.4% (Fig. S22b), indicating that capacitance control dominates the electrochemical reaction at high scan rates, contributing to the excellent rate performance [15].

To further investigate the diffusion coefficient of Zn^2+^ (${D}_{{Zn}^{2+}}$) between two electrodes, the GITT was employed. As shown in Figs. 3d, e and S23, the ${D}_{{Zn}^{2+}}$ value for KNVO-C_3_N_4_ (ranging from ~ 9.92 × 10^−9^ to 7.62 × 10^−7^ cm^2^ s^−1^ throughout the intercalation process) is significantly higher than that of KNVO (~ 10.33 × 10^−10^ to 8.06 × 10^−8^ cm^2^ s^−1^), NVO-C_3_N_4_ (~ 11.27 × 10^−11^ to 8.34 × 10^−8^ cm^2^ s^−1^), and NVO (~ 12.10 × 10^−12^ to 9.54 × 10^−9^ cm^2^ s^−1^). Compared to the intercalation of K^+^ or C_3_N_4_ alone, the synergistic effect of K^+^ and C_3_N_4_ co-intercalation more effectively enhances the ion diffusion kinetics of the electrode. In addition, it is found that the Zn^2+^ diffusion coefficient (Fig. S24) decreases with increasing C_3_N_4_ intercalation content, indicating the existence of an optimal C_3_N_4_ intercalation content. Moreover, the reaction kinetics of the electrodes were further conducted through EIS. Figure S25 shows that the charge-transfer resistance (R_ct_) of the KNVO-C_3_N_4_ electrode is lower than that of the NVO, KNVO, and NVO-C_3_N_4_ electrodes. It is noteworthy that while the intercalation of K^+^ or C_3_N_4_ alone does not decrease the R_ct_ as effectively as the co-intercalation of K^+^ and C_3_N_4_, it still facilitates charge transfer relative to NVO. Interestingly, with increased C_3_N_4_ intercalation content, R_ct_ has not decreased (Fig. S26), indicating that only an appropriate C_3_N_4_ intercalation content can further improve the reaction kinetics and the ion transfer rate. Excessive C_3_N_4_ intercalation is not conducive to the reaction kinetics of the electrode. Furthermore, changes in charge transfer during the first cycle were studied using ex situ EIS to explore the transport kinetics evolution of the KNVO-C_3_N_4_ electrode, as shown in Fig. 3f–h. During the initial discharge process, R_ct_ increases when the discharge reaches 0.8 V, primarily due to the dissolution of hydrated Zn^2+^ at the interface. As discharge continues to 0.2 V, R_ct_ gradually decreases due to the gradual activation of the cathode and the insertion of more Zn^2+^, suggesting that R_ct_ may be related to the Zn^2+^ content in the cathode. The rise in R_ct_ during charging to 0.4 V may be attributed to the interfacial tension generated by the extraction of Zn^2+^ from the interlayer. Upon charging to 1.6 V, R_ct_ sharply decreases due to the increased lattice distance of KNVO-C_3_N_4_ [15, 25]. Overall, the EIS results indicate that K^+^ and C_3_N_4_ co-intercalation effectively enhances the charge transfer kinetics.

### Theoretical Calculations

**Fig. 4:**
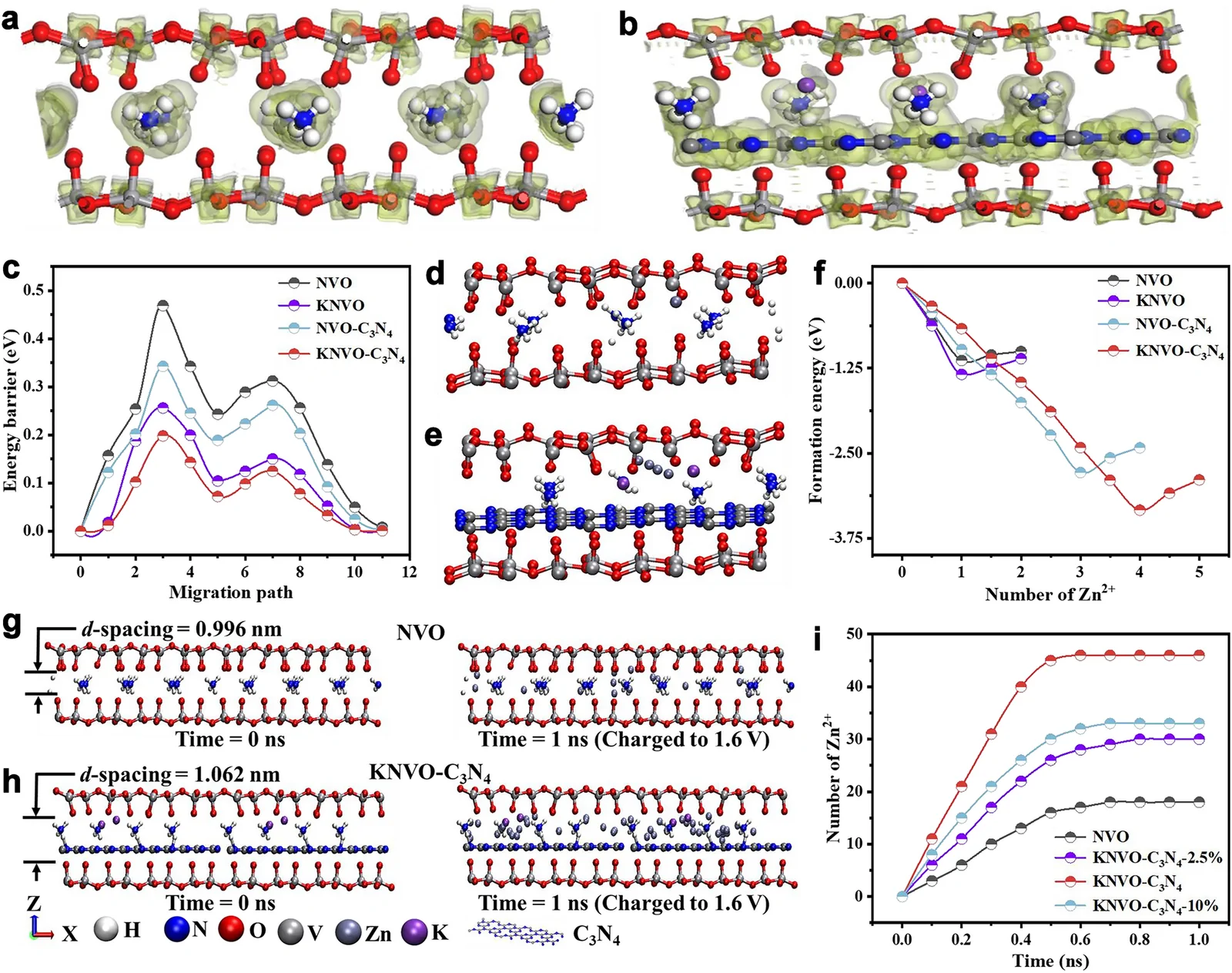
**a**,** b** Differential charge density with Zn^2+^ intercalation in NVO and KNVO-C_3_N_4_. **c** Calculated Zn^2+^ diffusion barriers in NVO, KNVO, NVO-C_3_N_4_, and KNVO-C_3_N_4_. **d**, **e** The schematic of the structure after insertion of Zn^2+^ into NVO and KNVO-C_3_N_4_. **f** Calculated Zn^2+^ insertion formation energy in NVO, KNVO, NVO-C_3_N_4_, and KNVO-C_3_N_4_. **g**, **h** MD simulation structures of ion diffusion through NVO and KNVO-C_3_N_4_ nanochannels. **i** Number evolution of Zn.^2+^ in the samples with different content of C_3_N_4_

To further explore the intrinsic effects of K^+^ and C_3_N_4_ co-intercalation on reaction kinetics, DFT calculations were conducted. Charge density difference analysis of NVO, KNVO, NVO-C_3_N_4_, and KNVO-C_3_N_4_ (Figs. 4a, b and S27) reveals the change in electronic structure following K^+^ and C_3_N_4_ co-intercalation. Compared to NVO, KNVO, and NVO-C_3_N_4_, KNVO-C_3_N_4_ exhibits a larger electron cloud, resulting in the weaker electrostatic interaction between the Zn^2+^ and [VO_n_] layer, which is more conducive to the improvement of diffusion kinetics [16]. Calculated diffusion paths (Fig. S28) and energy barriers (Fig. 4c) also show that the diffusion energy barrier of KNVO-C_3_N_4_ is significantly lower than that of NVO, KNVO, and NVO-C_3_N_4_. This suggests that the synergistic effect of K^+^ and C_3_N_4_ co-intercalation reduces the Zn^2+^ diffusion energy barrier, promoting Zn^2+^ diffusion and contributing to its excellent ultrahigh rate performance. In addition, to evaluate the maximum amount of Zn^2+^ insertion in NVO, KNVO, NVO-C_3_N_4_, and KNVO-C_3_N_4_ samples, formation energy calculations are performed, as shown in Figs. 4d–f and S29. The maximum Zn^2+^ intercalation amount of KNVO-C_3_N_4_ is 4.0, surpassing the other three samples, indicating a preference for Zn^2+^ embedding in KNVO-C_3_N_4_, thereby enhancing storage capacity [51]. The absolute values of formation energy (KNVO-C_3_N_4_ > NVO-C_3_N_4_ > KNVO > NVO) indicate that the KNVO-C_3_N_4_ structure is the most stable [52, 53]. Comparing NVO-C_3_N_4_, KNVO, and NVO reveals that C_3_N_4_ intercalation is the primary factor for improved structural stability, consistent with previous cycling stability results.

The above GITT results show that the interlayer spacing difference caused by different C_3_N_4_ contents in the intercalation leads to different Zn^2+^ diffusion coefficients of samples (NVO, KNVO-C_3_N_4_-2.5%, KNVO-C_3_N_4_, and KNVO-C_3_N_4_-10%), as shown in Figs. 3d and S24. To further elucidate the underlying reasons, molecular dynamics (MD) simulations were conducted on these samples. The MD models are depicted in Figs. 4g, h, and S30, with interlayer spacings calculated from XRD results (Fig. S3). As interlayer spacing increases, the Zn^2+^ number initially rises and then declines (Fig. 4i), suggesting that there is an optimal interlayer spacing during the charging process that enhances Zn^2+^ diffusion within the electrode. At a simulation time of 1 ns, the number of Zn^2+^ stabilizes. Notably, the slope of the Zn^2+^ number in KNVO-C_3_N_4_ is steeper than in the other three samples, indicating that it possesses the highest Zn^2+^ diffusion coefficient [54]. Based on the results from EIS, GITT, and MD simulations, we conclude that the increased interlayer spacing in KNVO-C_3_N_4_ facilitates Zn^2+^ transport and significantly reduces the electrostatic interaction between Zn^2+^ and the [VO_n_] layer, leading to enhanced Zn^2+^ mobility and accelerated kinetics. However, the sample with a higher C_3_N_4_ intercalation content (KNVO-C_3_N_4_-10%) has not further increased the Zn^2+^ diffusion coefficient; instead, it results in decreased kinetics. This phenomenon is attributed to the adverse effect of excessive intercalation of the poorly conductive C_3_N_4_ on the host material.

### Energy Storage Mechanism

**Fig. 5:**
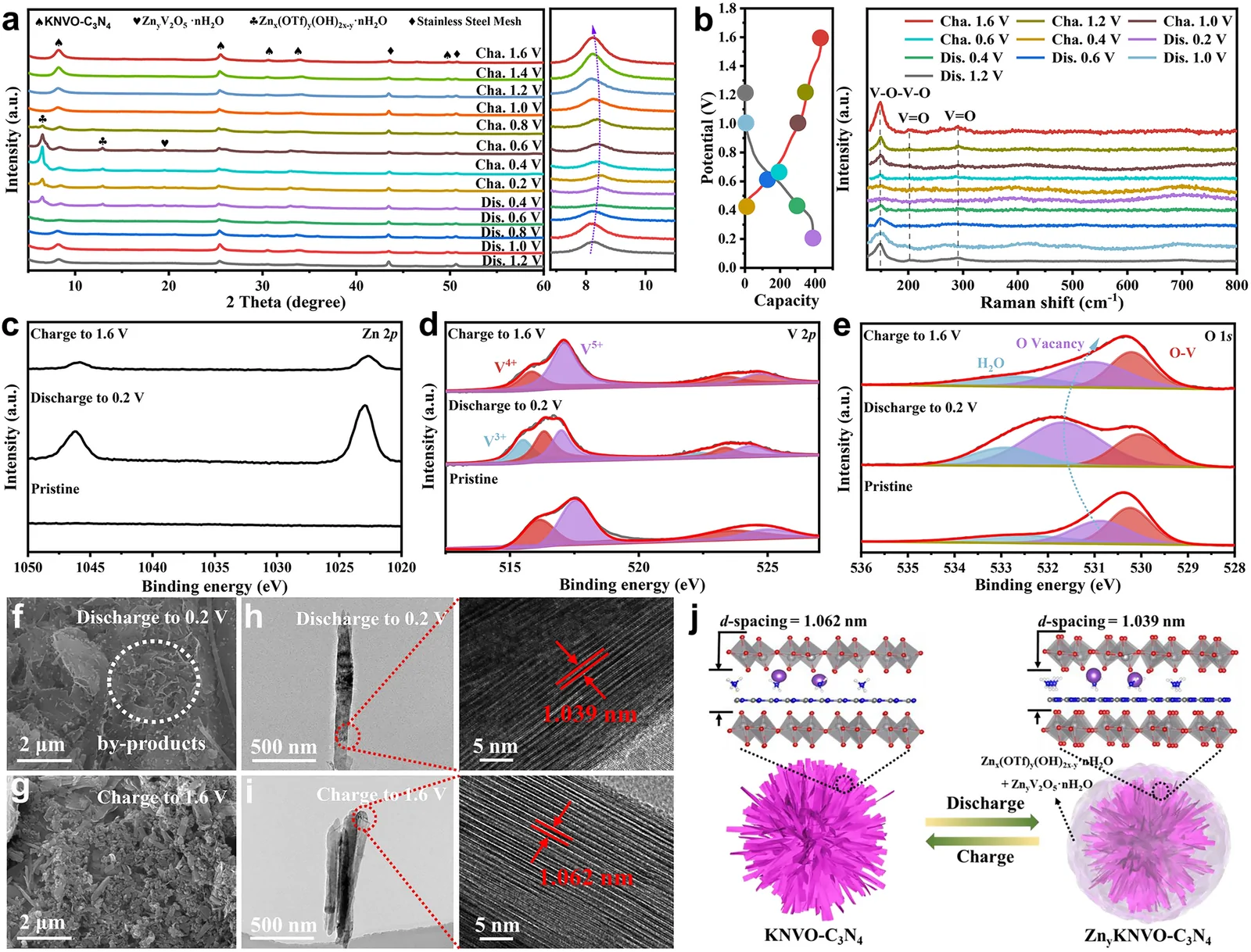
**a** Ex situ XRD patterns of the KNVO-C_3_N_4_ electrode at different voltage states during the first cycle of charge/discharge processes at 0.5 A g^−1^. **b** Ex situ Raman spectra of the KNVO-C_3_N_4_ electrode at different voltage states during the first cycle of charge/discharge processes at 0.5 A g^−1^. **c-e** The corresponding ex situ XPS spectra of Zn 2*p*, V 2*p*, and O 1*s*. **f-i** SEM, TEM, and HRTEM of the KNVO-C_3_N_4_ electrode at different states. **j** Schematic illustration of the reaction mechanism

To elucidate the Zn-ion storage mechanism of the KNVO-C_3_N_4_ electrode, multiple ex situ characterizations were employed to investigate the morphological and structural evolution during cycling. As shown in Fig. 5a, during the discharge process, the (001) phase in the ex situ XRD pattern shifts toward a higher angle, which can be attributed to the lattice contraction caused by the strong electrostatic interaction following Zn^2+^ insertion [20]. In the subsequent charging process, as Zn^2+^ is de-intercalated, the phase shift returns to its initial position, indicating that the KNVO-C_3_N_4_ electrode exhibits good reversibility. Notably, a new peak at 19.6° appears in the host material during the charge/discharge process, corresponding to the reversible Zn_y_V_2_O_5_·nH_2_O phase, mainly due to the co-existence of Zn_y_V_2_O_5_·nH_2_O and Zn_y_KNVO-C_3_N_4_ phases formed after the co-insertion of Zn^2+^ and H_2_O into the host material [42]. Besides, two new peaks located at 6.5° and 13.2°, which are attributed to the by-products (Zn_x_(OTf)_y_(OH)_2x−y_·nH_2_O) generated by the reaction of Zn(CF_3_SO_3_)_2_ in the electrolyte with OH^−^ produced by the decomposition of water [15, 55]. These new peaks disappear when charged to 1.0 V and show regular fluctuations, indicating the reversibility of the phase transition in the KNVO-C_3_N_4_ electrode. Figure S31 presents the ex situ XRD patterns of the KNVO-C_3_N_4_ electrode after different cycles, showing no significant changes in the peaks, which demonstrates its good cycling stability. Ex situ Raman spectroscopy (Fig. 5b) was then conducted, revealing the evolution of the V–O–V–O and V=O frameworks of the KNVO-C_3_N_4_ electrode during the charge/discharge process. During the Zn^2+^ intercalation, the intensity of V–O–V–O and V=O bonds decreases, indicating slight degradation of the V–O framework structure during charging [17]. However, during the Zn^2+^ de-intercalation, the intensity of bonds gradually recovers, further proving that the KNVO-C_3_N_4_ electrode has good reversibility in the electrochemical reaction.

Ex situ XPS spectra were carried out to explore the valence state changes in the KNVO-C_3_N_4_ electrode during the Zn^2+^ intercalation/de-intercalation process. As shown in Fig. 5c, no Zn 2*p* spectrum signal is detected in the original state. When discharges to 0.2 V, two strong characteristic peaks of Zn 2*p*_1/2_ (1045.2 eV) and Zn 2*p*_3/2_ (1022.1 eV) are detected, confirming the successful insertion of Zn^2+^ into the KNVO-C_3_N_4_ electrode [56]. When charges to 1.6 V, the intensity of the Zn 2*p* peak diminishes, indicating that most Zn^2+^ are removed from the KNVO-C_3_N_4_ electrode, with residual zinc ions possibly originating from Zn^2+^ in the crystal lattice or Zn^2+^ adsorbed on the electrode surface. In the V *2p* spectrum (Fig. 5d), compared to the original electrode, the intensities of V^5+^ and V^4+^ significantly decrease upon discharging to 0.2 V, and a new peak classified as V^3+^ appears at 515.3 eV, associated with the reduction in V during the Zn^2+^ intercalation [43]. Conversely, in the fully charged state, the valence state of V is restored, indicating a highly reversible redox reaction at the electrode [57]. Regarding the O 1*s* spectrum (Fig. 5e), it is observed that when discharged to 0.2 V, the H_2_O peak intensity increases, mainly due to the intercalation of H^+^ in the host material and the formation of bound water with oxygen, as well as the formation of Zn_x_(OTf)_y_(OH)_2x–y_·nH_2_O [58]. Interestingly, when fully charged, the H_2_O peak persists, indicating that the H_2_O molecules are not completely separated during the Zn^2+^ de-intercalation, supporting the co-intercalation mechanism of H_2_O molecules and Zn^2+^. Meanwhile, the binding energy of OVs shifts to higher values when discharged to 0.2 V and returns to its original position upon charging to 1.6 V. This shift to higher binding energy is likely attributable to the induction of numerous electron lone pairs by the OVs during the discharge process, changing the electronic arrangement [59]. Additionally, it can be seen from Fig. S32 that after the first cycle, the peak area of NH_4_^+^ weakens when discharged to 0.2 V, and nearly recovers upon charging to 1.6 V, indicating the highly reversible de/embedding of NH_4_^+^ with Zn^2+^ intercalation/de-intercalation.

Figure 5f–i shows the SEM, TEM, and HRTEM images of the KNVO-C_3_N_4_ electrode in different states. In the pristine and fully charged states, nanosheets are visible in the KNVO-C_3_N_4_ electrode, whereas nanosheet-like by-products (Zn_x_(OTf)_y_(OH)_2x-y_·nH_2_O) are observed on the surface in the fully discharged state, as shown in Figs. 5f, g and S33a [20]. Additionally, optical images (Fig. S33b) reveal that the aforementioned by-product appears as a white powder. Interestingly, ex situ HRTEM shows that the interlayer spacing of the (001) plane decreases to 10.39 Å in the fully discharged state and recovers in the fully charged state, consistent with the above ex situ XRD results. This phenomenon further confirms the good reversible structure of the electrode material. Based on these findings, the energy storage mechanism of the KNVO-C_3_N_4_ electrode is illustrated in Fig. 5j. When fully discharged, the intercalation of Zn^2+^ leads to a reduction in interlayer spacing and NH_4_^+^ content in the electrode material. When fully charged, the interlayer spacing and NH_4_^+^ content gradually recover, accompanied by the gradual disappearance of the by-product. These results demonstrate the effectiveness of K^+^ and C_3_N_4_ co-intercalation in enhancing the structural stability.

### Electrochemical Performance of the Pouch Cell at Extreme Temperatures

**Fig. 6:**
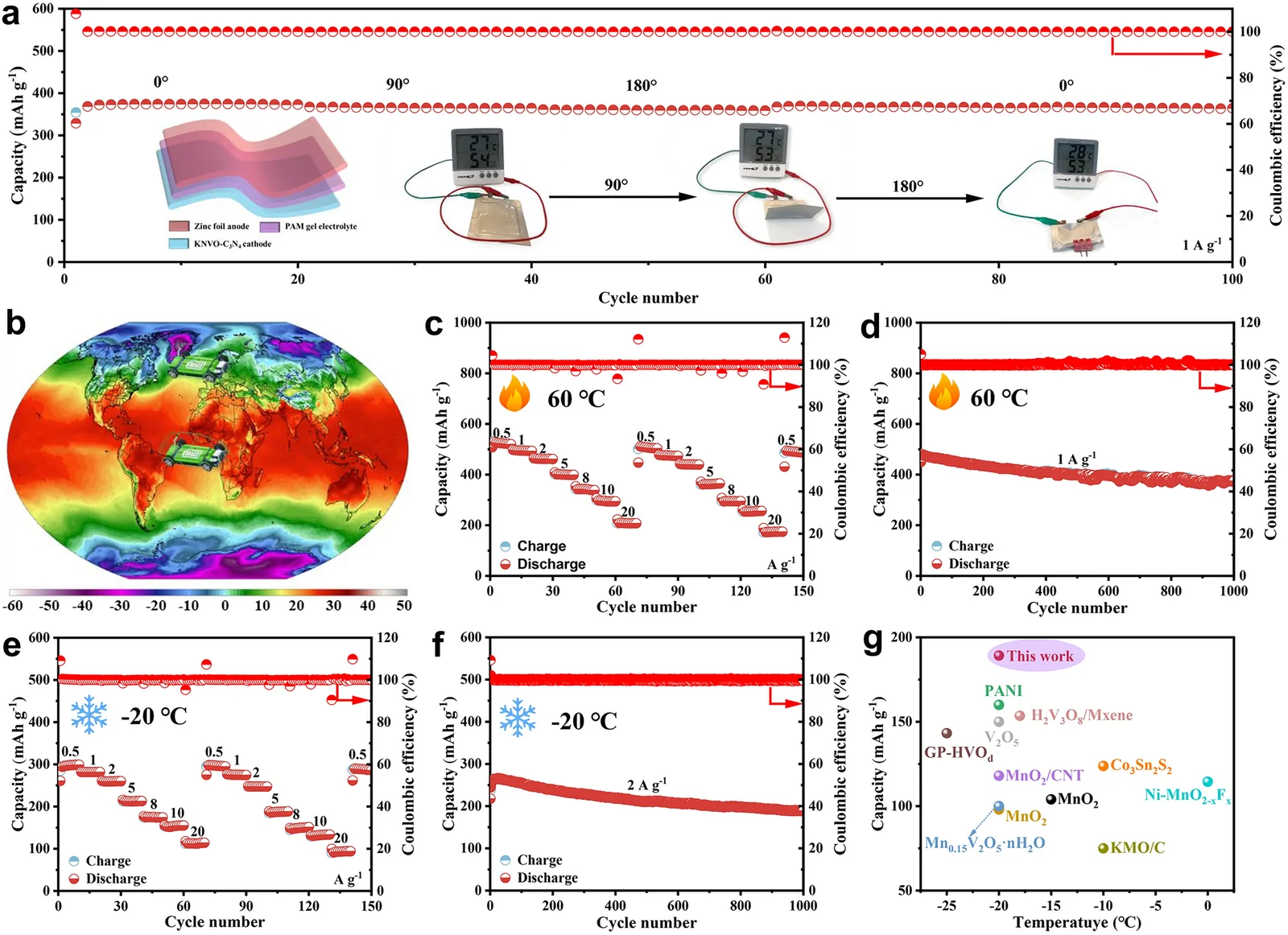
**a** The cycling performance of the pouch cell at the current density of 1 A g^−1^ under multiple bending at room temperature (The illustrations are schematic diagrams of the pouch cell and the thermometer working at different bending angles, respectively.) **b** Global temperature distribution on Dec. 1, 2024 (Image from Climate Reanalyzer, Climate Change Institute, University of Maine, USA). **c, d** Rate performance and long-term cycling stability of pouch cell at 60 °C. **e, f** Rate performance and long-term cycling stability of pouch cell at − 20 °C. **g** Comparison of the capacity of pouch cells and other reported batteries at low temperatures

To explore the potential application of the KNVO-C_3_N_4_ electrode in electronic devices, the pouch cells were assembled (as illustrated in Fig. 6a). Figure S34a shows that the specific capacity of the pouch cell ranges from 390.8 to 200.1 mAh g^−1^ as the current density increases from 0.5 to 20 A g^−1^. Upon reverting the current density to 0.5 A g^−1^, the specific capacity remains at 381.5 mAh g^−1^, exhibiting excellent reversibility. Compared to previously reported literature (Fig. S35), the pouch cell exhibits superior rate performance, indicating that it has good potential for practical applications. Even during long-term cycling stability tests at various currents (Figs. S34b and S36), the pouch cell shows outstanding cycling stability. Notably, it maintains a specific capacity of 168.7 mAh g^−1^ after 10,000 cycles at a current density of 10 A g⁻^1^, with a capacity retention rate of 66.0%. Additionally, the pouch cell also maintains good cycling stability at bending angles of 0°, 90°, and 180°, and can reliably power a thermometer at different bending angles, as shown in Figs. 6a and S37.

Considering the diversity of global climate (Fig. 6b), batteries operating in extreme environments can aggravate the instability of electrode materials and accelerate the rapid decay and degradation of battery performance. Therefore, developing V-based cathode materials with high performance across a wide temperature range is crucial. To assess the applicability of the KNVO-C_3_N_4_ electrode, its electrochemical performance was further evaluated under extreme temperature conditions. Figs. 6c–f displays the rate performance and the long-term cycling stability at −20 and 60 °C, respectively. Surprisingly, the pouch cell exhibits impressive electrochemical performance in extreme environments. Specifically, at a high current density of 20 A g^−1^, the electrode achieves high specific capacities of 111.3 and 208.6 mAh g^−1^ at −20 and 60 °C, respectively, indicating that the KNVO-C_3_N_4_ electrode maintains high reversibility even at extreme temperatures. Impressively, the KNVO-C_3_N_4_ electrode also exhibits significant advantages in electrochemical performance, outperforming most reported cathodes for pouch cell, as shown in Fig. 6g [40, 44, 60–68]. To elucidate the factors contributing to the excellent performance of pouch cells in extreme environments, both ionic conductivity and low-temperature resistance were evaluated at various temperatures. EIS results (Fig. S38a, b) reveal that impedance decreases with increasing temperature, indicating enhanced ion migration at elevated temperatures. The ionic conductivity values are 20.56 mS cm^−1^ at 60 °C and 2.30 mS cm^−1^ at −20 °C (see the Supporting Information for calculation details), suggesting improved reaction kinetics at higher temperatures. Notably, the ionic conductivity remains as high as 2.30 mS cm^−1^ at −20 °C, demonstrating excellent low-temperature tolerance and providing efficient ion transport pathways for Zn^2^⁺ ions [69]. Furthermore, the calculated activation energy (${E}_{a}$) is 6.91 kJ mol^−1^ (Fig. S38c), indicating rapid ion diffusion even at low temperatures and confirming that the KNVO-C₃N₄ cathode exhibits favorable reaction kinetics under extreme environmental conditions [70, 71]. Therefore, based on these results, the KNVO-C_3_N_4_ electrode material achieves excellent electrochemical performance across a wide temperature range of −20 to 60 °C, highlighting its substantial application potential.

## Conclusion

In summary, K^+^ and C_3_N_4_ co-intercalated NVO nanosheets (KNVO-C_3_N_4_) with adjustable interlayer spacing were synthesized using a straightforward method. The intercalation of C_3_N_4_ enhances the structural stability, effectively preventing the structural collapse of the NVO during prolonged cycling. In addition, KNVO-C_3_N_4_ exhibits high Zn^2+^ diffusion kinetics and fast charge transfer kinetics, which is mainly attributed to its adjustable interlayer spacing and the synergistic effect of K^+^ co-intercalation with optimal C_3_N_4_ content. Furthermore, the Zn^2+^ storage mechanism of the KNVO-C_3_N_4_ cathode is revealed through ex situ characterization techniques (XRD, Raman, XPS, SEM, and TEM), showcasing its excellent structural stability and reversibility during the charge/discharge process. As expected, the KNVO-C_3_N_4_ cathode exhibits excellent rate performance, outstanding long-term cycling stability, and high power/energy density at room temperature. Notably, even under extreme environments of -20 and 60 °C, it delivers high specific capacities of 111.3 and 208.6 mAh g^−1^ at a high current density of 20 A g^−1^, respectively. This work is expected to provide new insights into increasing the electrochemical performance of V-based materials through synergistic effects or interlayer spacing adjustment, thereby advancing the development of high-performance zinc-ion batteries in extreme environments.

## Supplementary Information

Below is the link to the electronic supplementary material.

Supplementary file 1 (DOCX 19 kb)
