# Supplementary material for: High-Performance Wide-Temperature Zinc-Ion Batteries with K+/C3N4 Co-Intercalated Ammonium Vanadate Cathodes
Source: Nanomicro Lett. 2025 Sep 1;18:48. doi: 10.1007/s40820-025-01892-0 (PMC12401862; doi:10.1007/s40820-025-01892-0)
Supplement: Supplementary file 1 — (DOCX 19 kb) [file 40820_2025_1892_MOESM1_ESM.docx]

Supporting Information for

**High-Performance Wide-Temperature Zinc-Ion Batteries with K^+^/C_3_N_4_ Co-Intercalated Ammonium Vanadate Cathodes**

Daming Chen^1^, [Jimin](https://research.polyu.edu.hk/en/persons/4793fffe-f28a-4f6f-b06f-66ff4223cad7) Fu^1^, Yang Ming^1^, Wei Cai^1^, Yidi Wang^2^, Xin Hu^1^, Rujun Yu^1^, Ming Yang^3^, Yixin Hu^1^, Benjamin Tawiah^1^, Shuo Shi^1^, Hanbai Wu^1^, Zijian Li^1^, Bin Fei^1^*

^1^Materials Synthesis and Processing Lab, School of Fashion and Textiles, The Hong Kong Polytechnic University, Kowloon, Hong Kong SAR 999077, P. R. China

^2^Department of Applied Biology and Chemical Technology, The Hong Kong Polytechnic University, Kowloon, Hong Kong SAR 999077, P. R. China

^3^College of Chemistry and Environmental Engineering, Shenzhen University, Shenzhen 518060, P. R. China

*Corresponding author. E-mail: [bin.fei@polyu.edu.hk](mailto:bin.fei@polyu.edu.hk) (Bin Fei)

## S1 Theoretical calculation

**Density Functional Theory (DFT) Calculations (For the Method Part)**

To investigate the structural stability and ion migration behavior in the NVO system with potassium (K^+^) ion intercalation and C_3_N_4_ interlayers, first-principles calculations were performed using the Quickstep module in the CP2K software package [S1] at the PBE-D3 dispersion-corrected functional level. The formation energy ($E_{Form}$) of the K^+^-intercalated C_3_N_4_ hybrid structure was calculated to evaluate its thermodynamic stability, while the diffusion energy barrier of Zn^2+^ ions within the interlayer channels was determined using the Nudged Elastic Band (NEB) method [S2]. The formation energy was defined as:

$E_{Form}= E_{Total}- E_{NVO}- E_{Zn}$ or $E_{Form}- E_{Total}- E_{KNVO-C3N4}- E_{Zn}$

Where $E_{Total}$ is the total energy of the hybrid system, $E_{KNVO-C3N4}$ is the energies of the NVO or KNVO-C_3_N_4_ system without inserting Zn ion, respectively, and $E_{Zn}$ is the energy of an isolated Zn ion. The results revealed a negative formation energy of KNVO-C_3_N_4_, indicating the spontaneous stabilization of the hybrid structure.

For Zn^2+^ diffusion, the NEB method was employed to locate the minimum energy path (MEP) between initial and final states. A 2×2×1 supercell was used to model the NVO or KNVO-C_3_N_4_ system, and the climbing image algorithm ensured accurate transition state identification. The calculated energy barrier for Zn^2+^ migration through the K ion and C_3_N_4_-modified interlayer was 0.198 eV, significantly lower than that in pristine NVO (0.469 eV), suggesting enhanced ion mobility due to the expanded interlayer spacing and electronic coupling effects induced by K ion and C_3_N_4_.

The calculations utilized Goedecker-Teter-Hutter (GTH) pseudopotentials [S3] with MOLOPT basis sets for all atoms. A plane-wave cutoff energy of 400 Ry was applied, and the DZVP basis set was adopted for geometry optimizations. Structural relaxations were performed until residual forces on all atoms were below 0.01 eV Å^−1^. The NEB calculations were implemented via the CP2K interface with the ASE (Atomic Simulation Environment) toolkit [S4], ensuring compatibility with climbing image optimization.

**MD simulation model and simulation setting details (For the Method Part)**

The NVO/KNVO-C_3_N_4_ cathode were modeled as a NVO layer system with pre-exist ion and C_3_N_4_ interlayer. The simulation system employed periodic boundary conditions in the X and Y directions, with an orthorhombic cell volume of 75×75×10 Å³. To describe interactions between Zn²⁺, CF_3_SO_3_⁻, K^+^ ions, and other components, the Optimized Potentials for Liquid Simulations All Atom (OPLS-AA) force field was utilized [S5]. Water molecules were modeled using the SPC/E rigid water potential [S6], while the Universal Force Field (UFF) [S7] was applied to describe the Zn foil structure.

All simulations were performed using the LAMMPS package [S8] under the NVT ensemble (constant number of particles, volume, and temperature) with a target temperature of 300 K maintained via a Nosé-Hoover thermostat. A time step of 1 fs was adopted to ensure numerical stability. Short-range Van der Waals interactions were truncated at 12 Å, while Coulombic interactions were calculated with a cutoff of 18 Å. Long-range electrostatic forces were treated using the particle-particle particle-mesh (PPPM) algorithm to ensure accuracy. Prior to production runs, energy minimization and equilibration steps were conducted to relax the system.

## Calculation Details

The ionic conductivity ($\sigma$) was calculated according to the following formula [S9]:

$\sigma=\frac{l}{R_{ct}S}$ (S1)

where $R_{ct}$ represents the resistance according to EIS measurement, $l$ represents the thickness (30 μm), and $S$ is the PAM gel electrolyte area (2.0096 cm^-2^).

The corresponding activation energy according to the following Eqs. S2 and S3 [10]:

$\sigma=\frac{A}{T}e^{-\frac{E_{a}}{Tk}}$ (S2)

$In\left( \sigma T \right)=In\left( A \right)-\frac{E_{a}}{Tk}$ (S3)

where $A$, $E_{a}$, $k$, and $T$ are the Arrhenius constant, activation energy, Boltzmann constant, and absolute temperature, respectively.

**S2 Supplementary Figures and Tables**


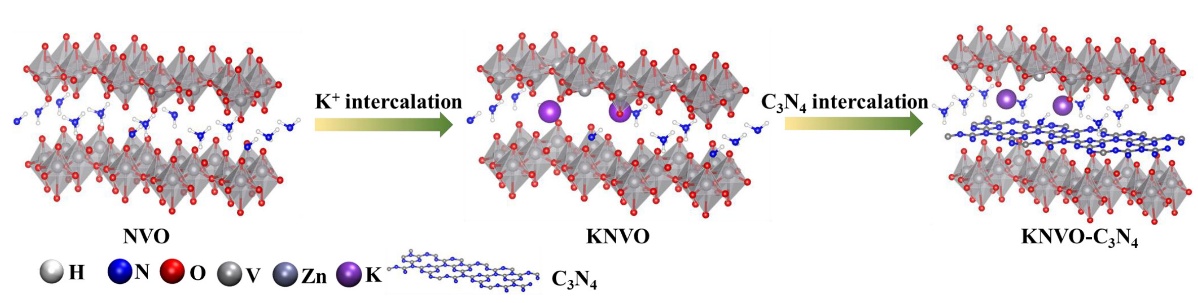


**Fig. S1** Schematic diagram of the preparation process of KNVO-C_3_N_4_

**Fig. S2** XRD pattern of C_3_N_4_

From the XRD test results, there are two obvious diffraction peaks at about 13.0° and 27.4°, indicating that the typical C_3_N_4_ is successfully synthesized [S11].


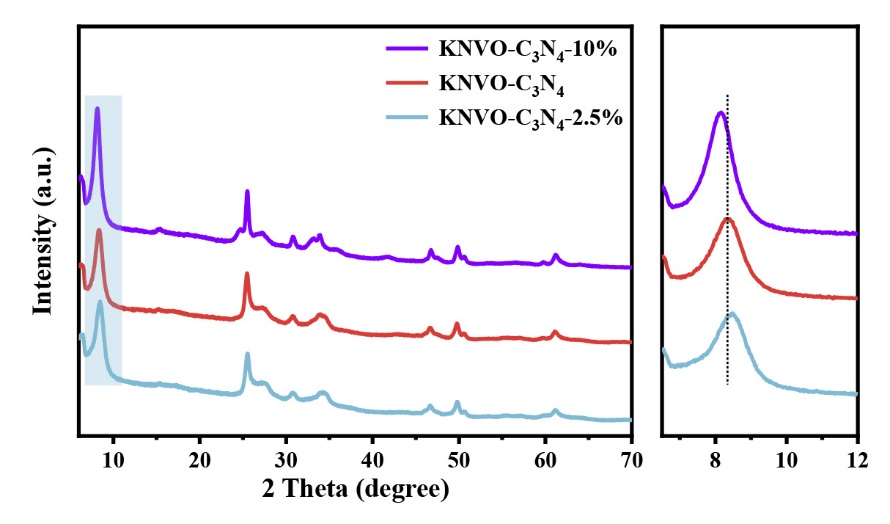


**Fig. S3** XRD pattern of KNVO-C_3_N_4_-2.5%, KNVO-C_3_N_4_ and KNVO-C_3_N_4_-10%

**Fig. S4** Tpical FTIR spectrum of C_3_N_4_

There are five obvious characteristic bands in the region of 1200-1700cm^-1^, which can be attributed to the typical stretching modes of C=N and C-N heterocycles in C_3_N_4_ [S12].

**Fig. S5** Raman spectra of NVO, KNVO, NVO-C_3_N_4_ and KNVO-C_3_N_4_


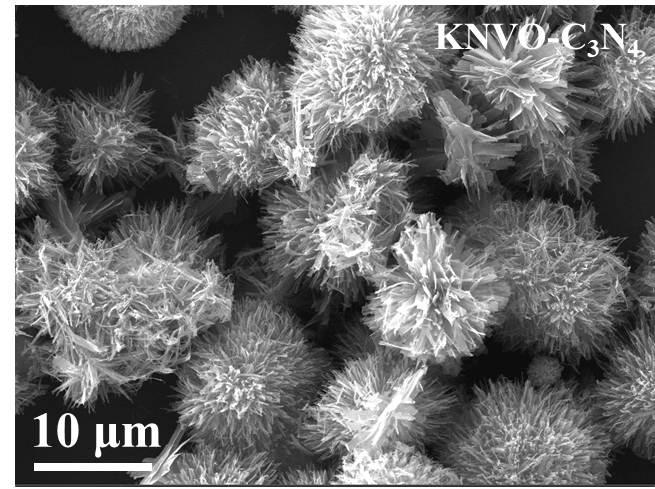


**Fig. S6** SEM image of KNVO-C_3_N_4_


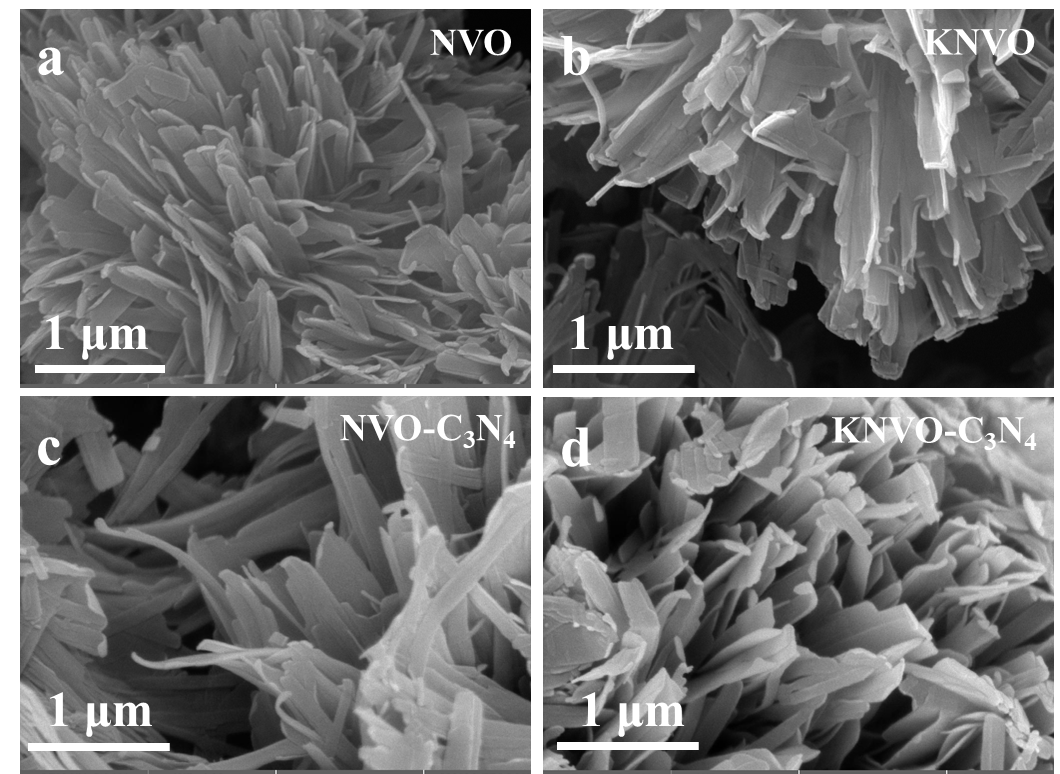


**Fig. S7** **a-d** SEM images of NVO, KNVO, NVO-C_3_N_4_ and KNVO-C_3_N_4_


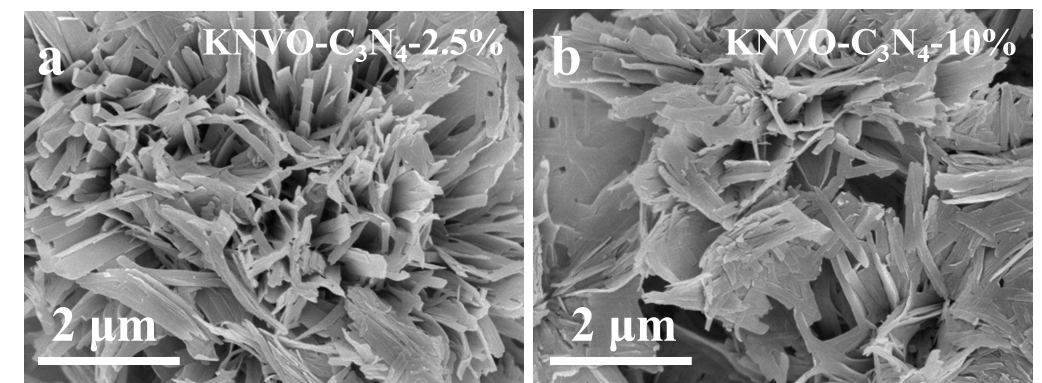


**Fig. S8** **a**, **b** SEM images of KNVO-C_3_N_4_-2.5% and KNVO-C_3_N_4_-10%, respectively


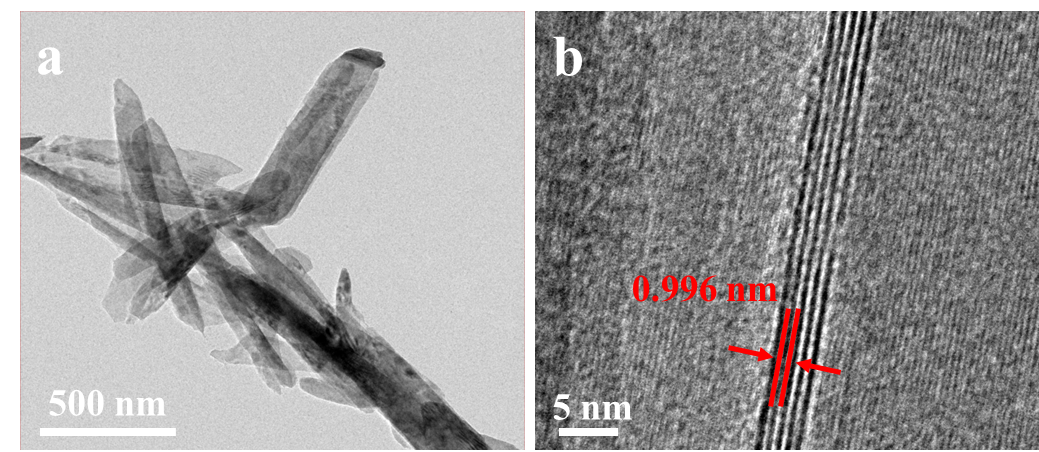


**Fig. S9** **a**, **b** TEM and HRTEM images of NVO


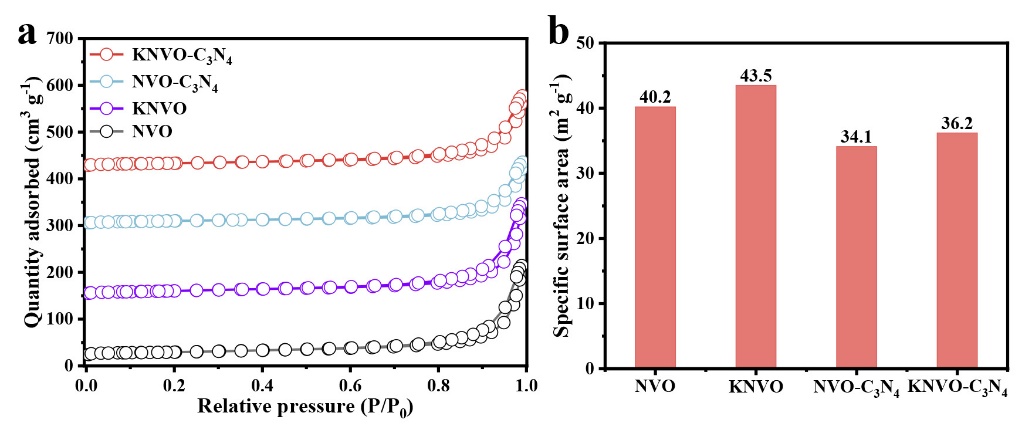


**Fig. S10** **a** Nitrogen adsorption and desorption plots of NVO, KNVO, NVO-C_3_N_4_ and KNVO-C_3_N_4_. **b** Specific surface area of NVO, KNVO, NVO-C_3_N_4_ and KNVO-C_3_N_4_

**Fig. S11** XPS spectra of NVO, KNVO, NVO-C_3_N_4_, and KNVO-C_3_N_4_


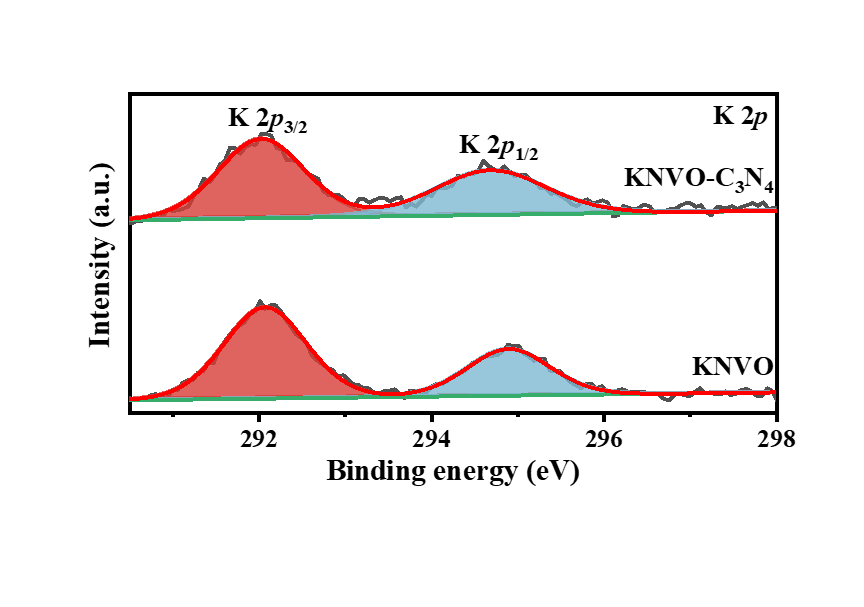


**Fig. S12** High-resolution XPS spectra of K 2*p*

**Fig. S13** High-resolution XPS spectra of N 1*s*

**Fig. S14** The initial three CV curves of NVO electrode recorded at 0.2 mV s^-1^


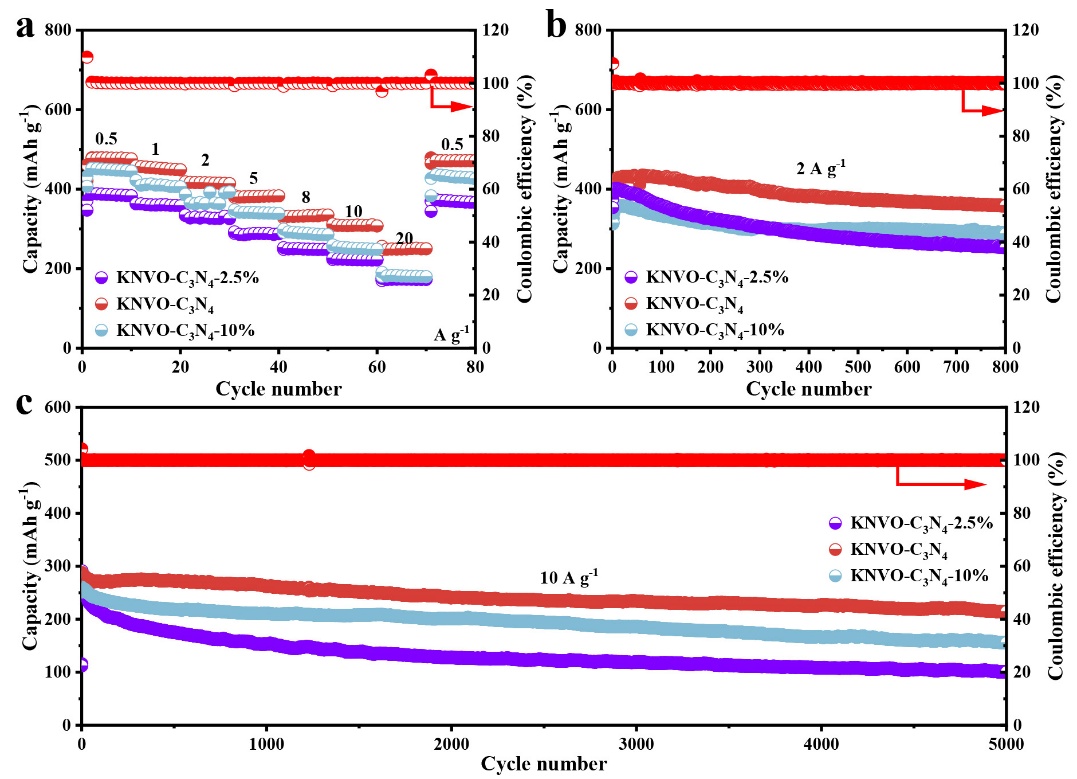


**Fig. S15** **a-c** Rate performances and cycling performances of KNVO-C_3_N_4_-2.5%, KNVO-C_3_N_4_, and KNVO-C_3_N_4_-10%


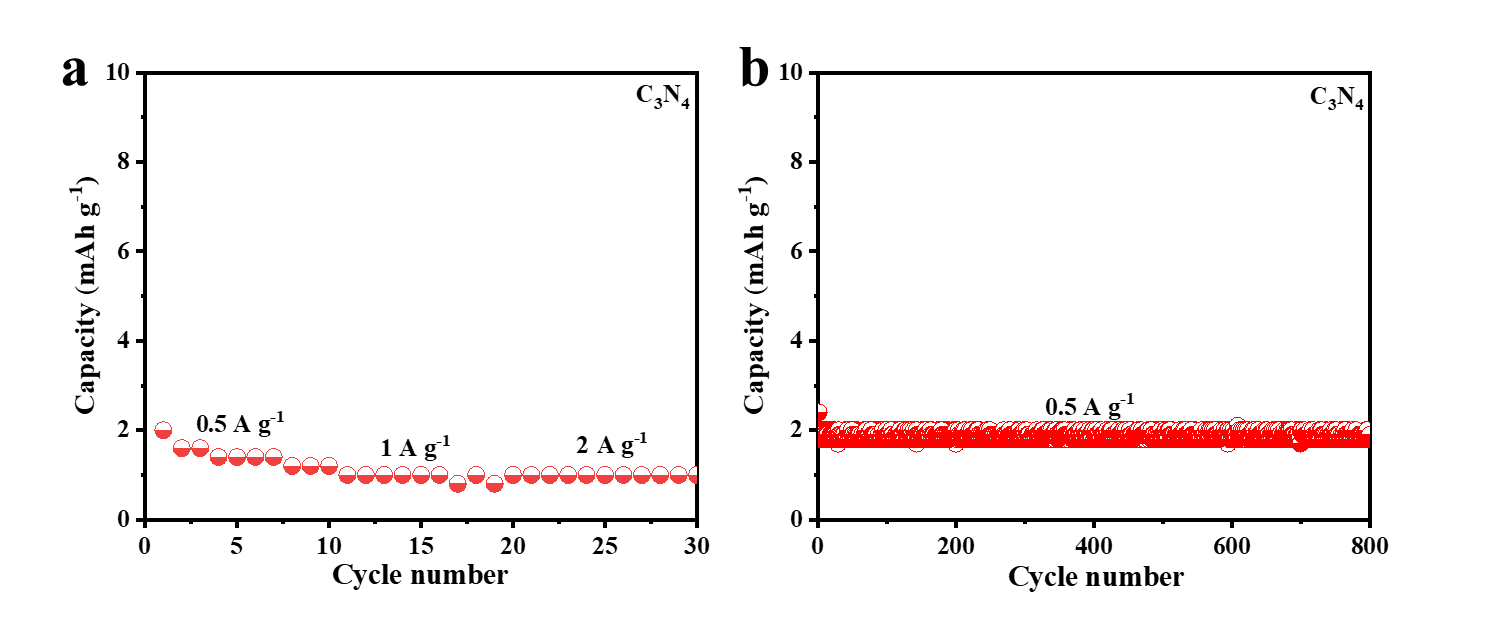


**Fig. S16** **a**, **b** Rate performances and cycling performances of C_3_N_4_


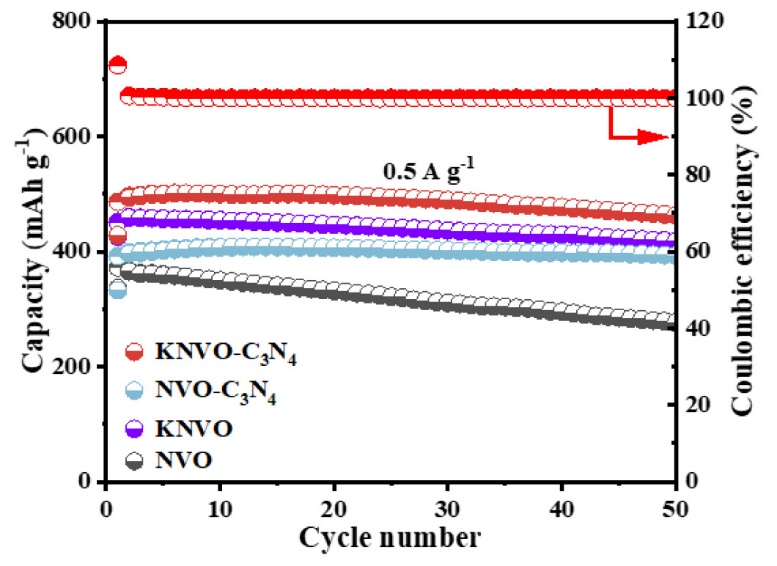


**Fig. S17** Cycling performances of NVO, KNVO, NVO-C_3_N_4_, and KNVO-C_3_N_4_ at a current density of 0.5 A g^-1^


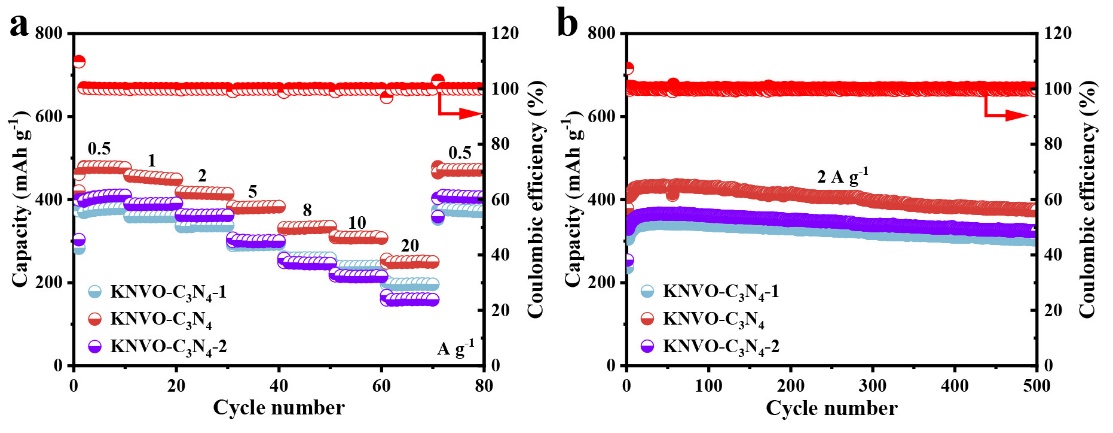


**Fig. S18** **a**, **b** Rate performances and cycling performances of KNVO-C_3_N_4_-1, KNVO-C_3_N_4_, and KNVO-C_3_N_4_-2


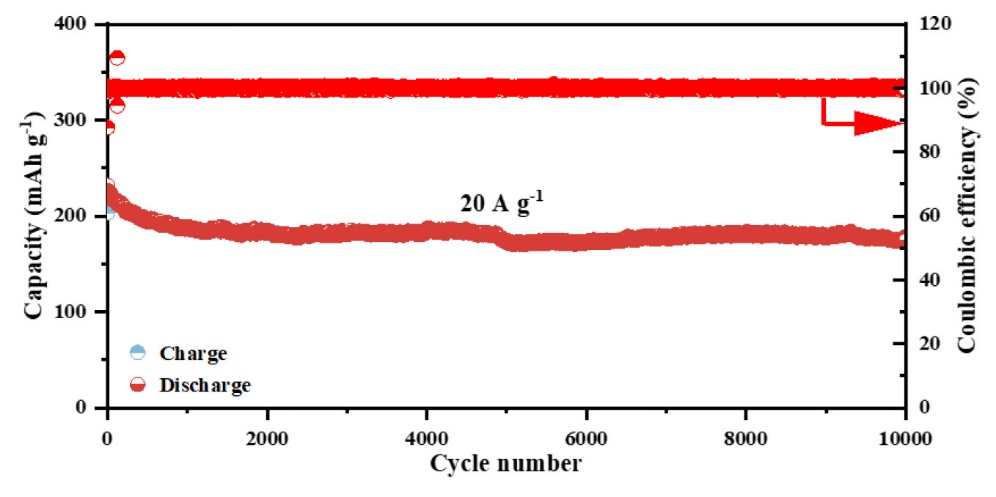


**Fig. 19** Long-cycling performance of KNVO-C_3_N_4_ at a current density of 20 A g^-1^.


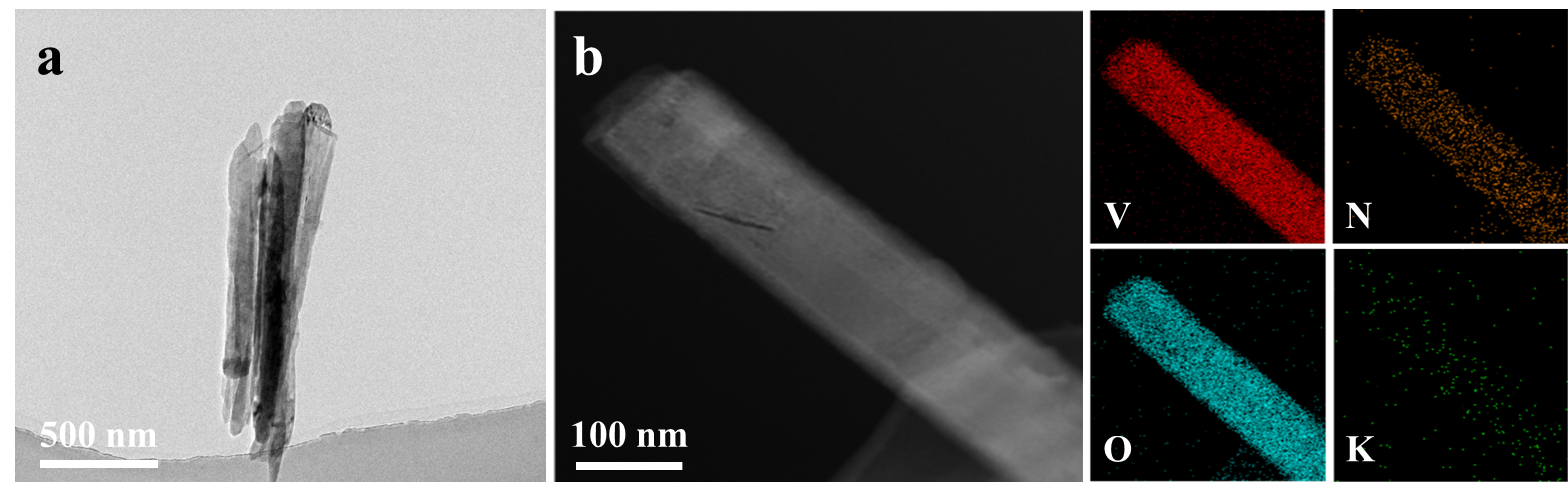


**Fig. S20 a** TEM image of KNVO-C_3_N_4_ after 100 cycles and **b** Corresponding HAADF-STEM image and the elemental distribution of V, N, O, and K


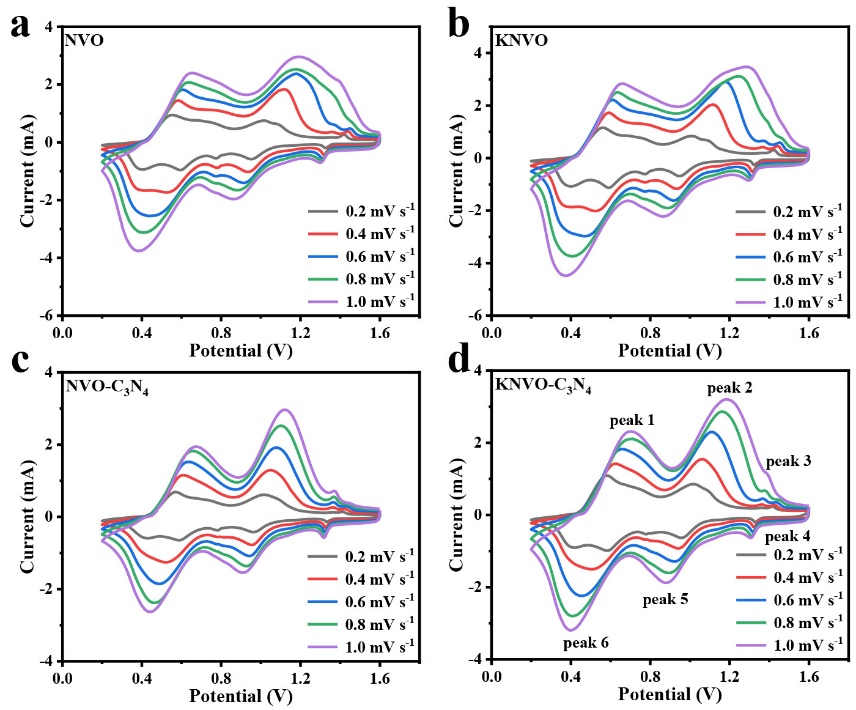


**Fig. S21** **a-d** CV curves of NVO, KNVO, NVO-C_3_N_4_, and KNVO-C_3_N_4_ at different scan rates


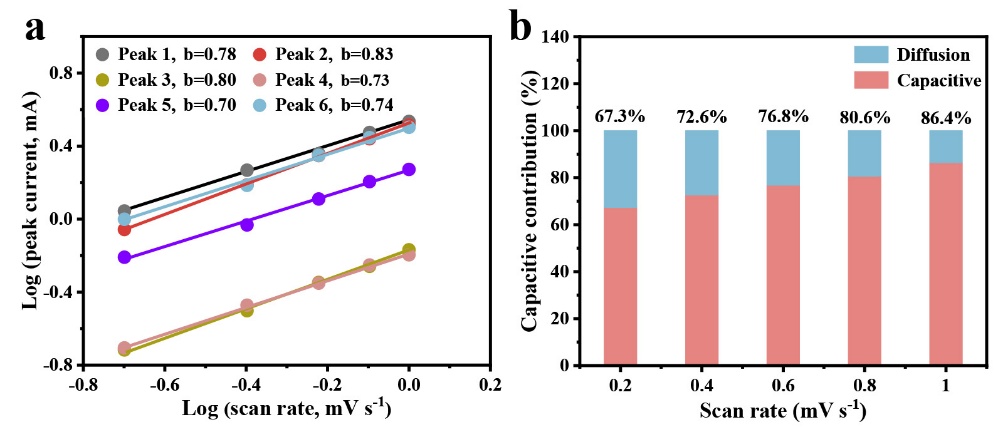


**Fig. S22** **a** Log(peak current) vs. log(scan rate) plots for the oxidation and reduction states of KNVO-C_3_N_4_ electrode. **b** Ratio of capacitive contribution in KNVO-C_3_N_4_ at different scan


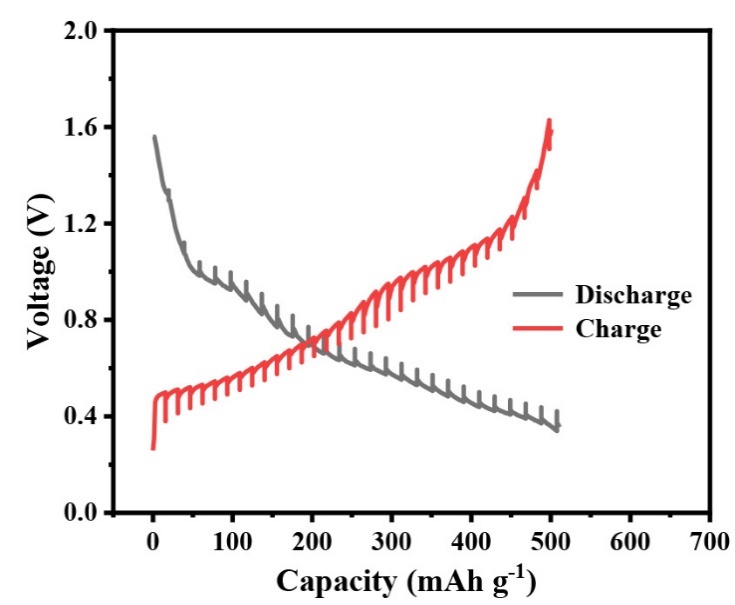


**Fig. S23** GITT potential profiles of KNVO-C_3_N_4_

The diffusion coefficient ($D_{{Zn}^{2+}}$) of NVO, KNVO, NVO-C_3_N_4_, and KNVO-C_3_N_4_ electrodes can be calculated according to the formula [S13]:

$D_{{Zn}^{2+}}=\frac{4}{\pi\tau}\left( \frac{m_{B}V_{M}}{M_{B}S} \right)^{2}{(\frac{{\triangle E}_{S}}{\triangle E_{\tau}})}^{2}$ (S4)

where $\tau$ denotes the duration time of the current pulse; $m_{B}$ and $V_{M}$ signify the mass of the active materials and the molar volume of the samples, respectively; $M_{B}$ and $S$ refer to the molecular weight and the total surface electrode in contact with the electrolyte, respectively; ${\triangle E}_{S}$ pertains to the change of steady-state voltage for the corresponding step; $\triangle E_{\tau}$ represents the potential difference during current pulse.


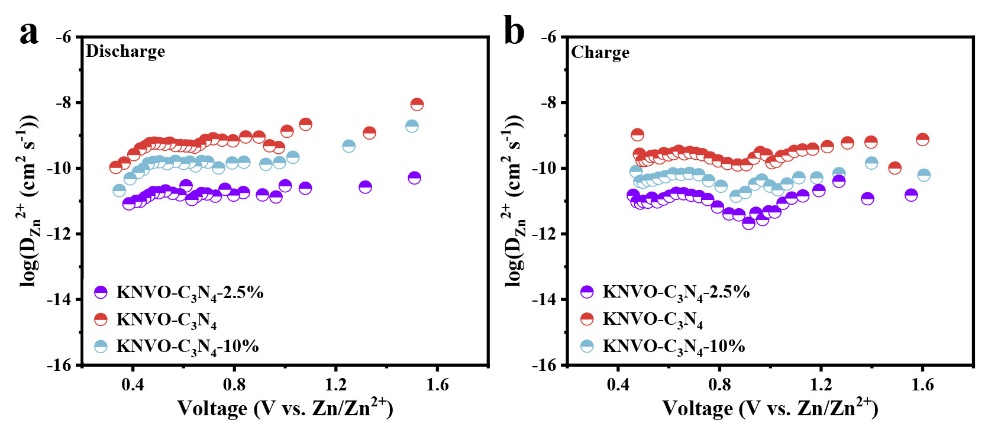


**Fig. S24** **a**, **b** Zn^2+^ diffusion coefficients versus different discharge/charge states

**Fig. S25** EIS curves of NVO, KNVO, NVO-C_3_N_4_, and KNVO-C_3_N_4_ (the inserted figure is the equivalent circuit model)


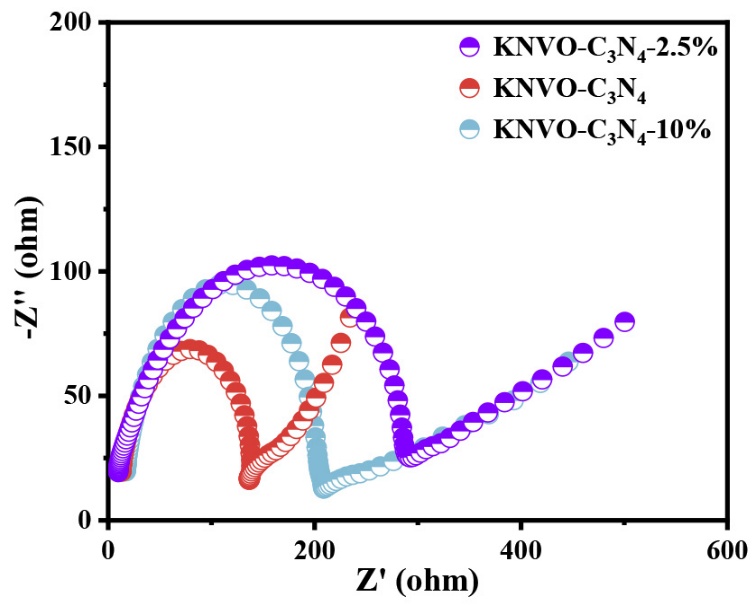


**Fig. S26** EIS curves of KNVO-C_3_N_4_-2.5%, KNVO-C_3_N_4_, and KNVO-C_3_N_4_-10%


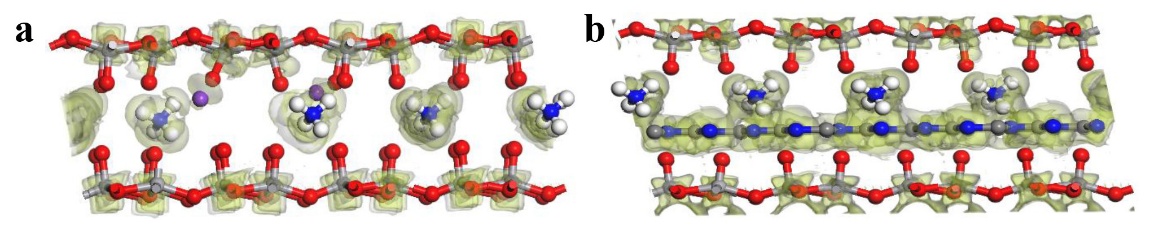


**Fig. S27** **a**, **b** Differential charge density with Zn^2+^ intercalation in KNVO and NVO-C_3_N_4_


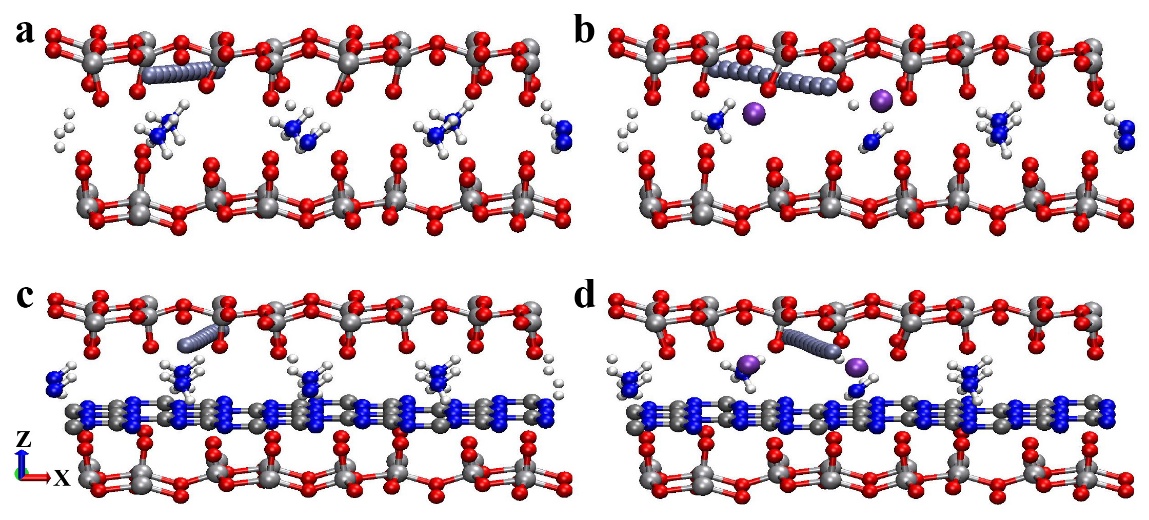


**Fig. S28** **a-d** Possible migration pathways for Zn^2+^ in NVO, KNVO, NVO-C_3_N_4_, and KNVO-C_3_N_4_


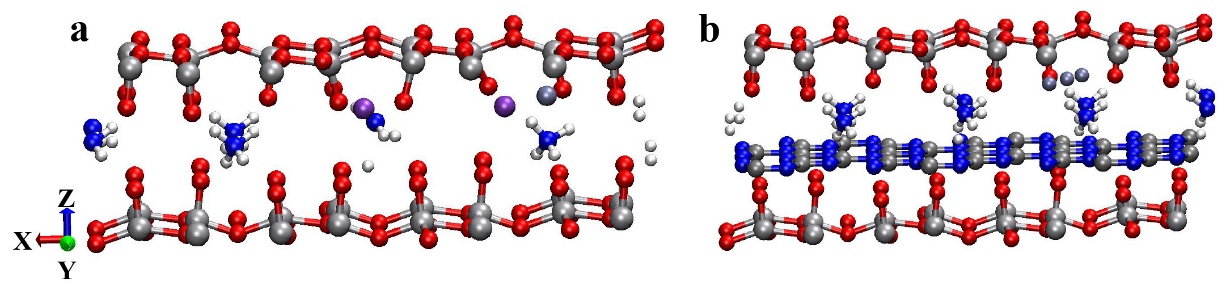


**Fig. S29** **a**, **b** The schematic of structure after insertion of Zn^2+^ into KNVO and NVO-C_3_N_4_


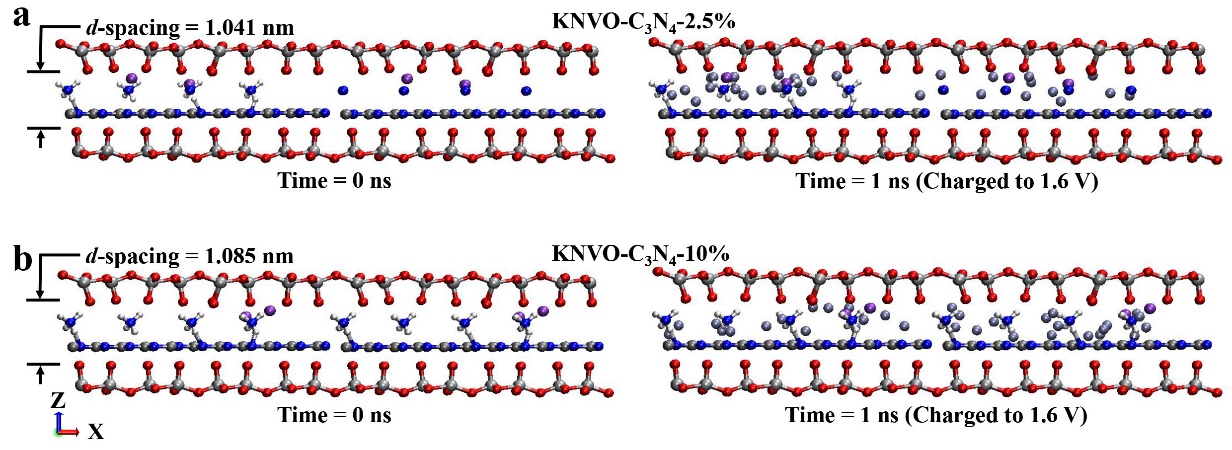


**Fig. S30** **a**, **b** Snapshots of Charging process: MD simulation structures of ion diffusion through KNVO-C_3_N_4_-2.5% and KNVO-C_3_N_4_-10% nanochannels


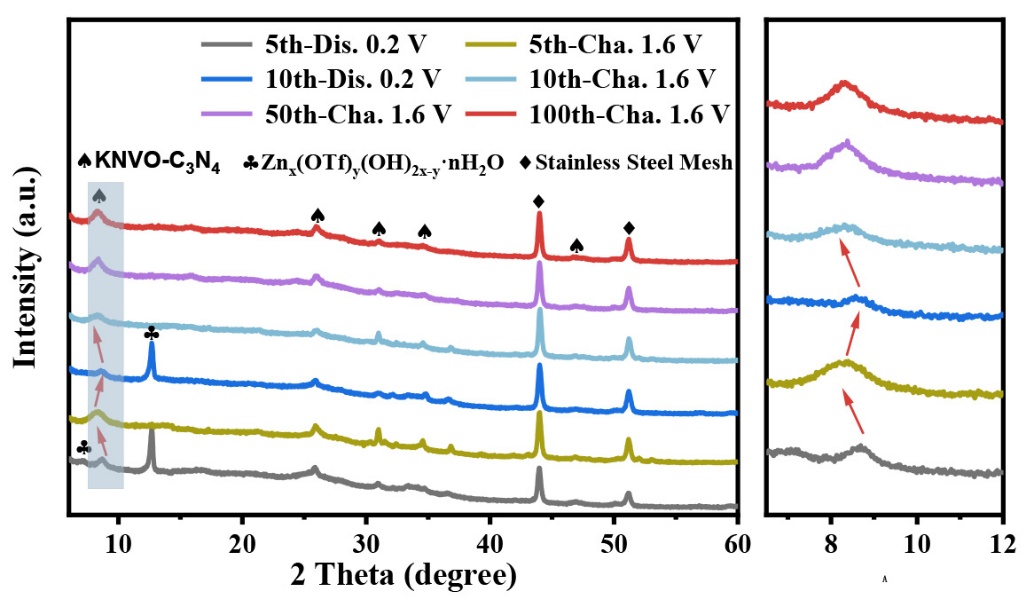


**Fig. S31** XRD patterns of the KNVO-C_3_N_4_ electrode at different cycles at a current density of 0.5 A g^-1^


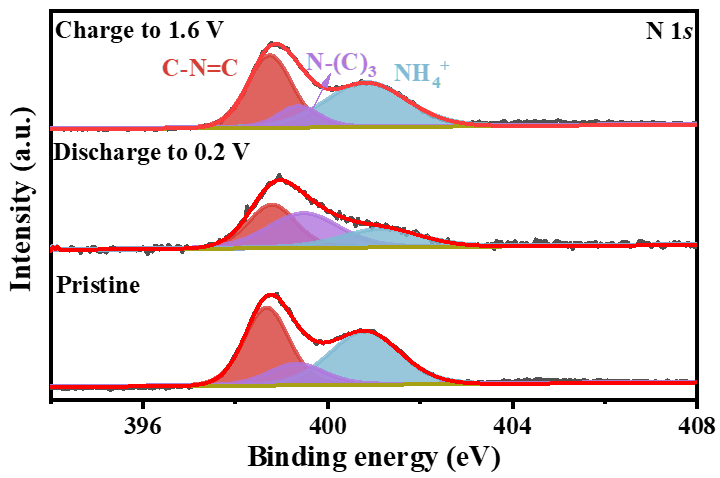


**Fig. S32** The corresponding ex-situ XPS spectra of N 1s


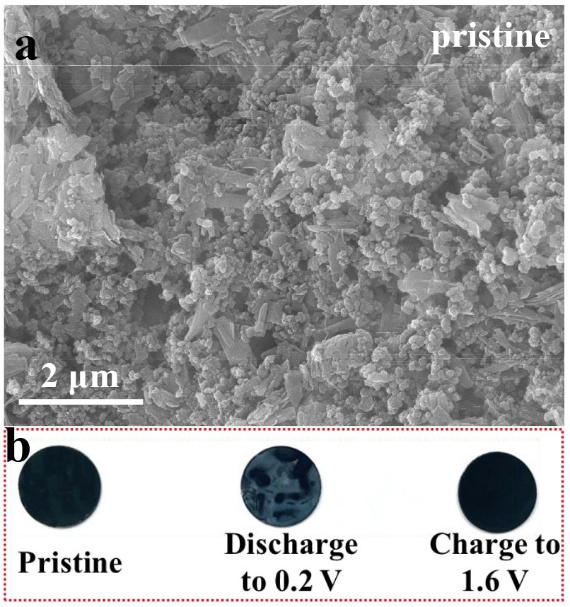


**Fig. S33** **a** SEM image of pristine KNVO-C_3_N_4_ electrode. **b** Electrode plate surface at different state


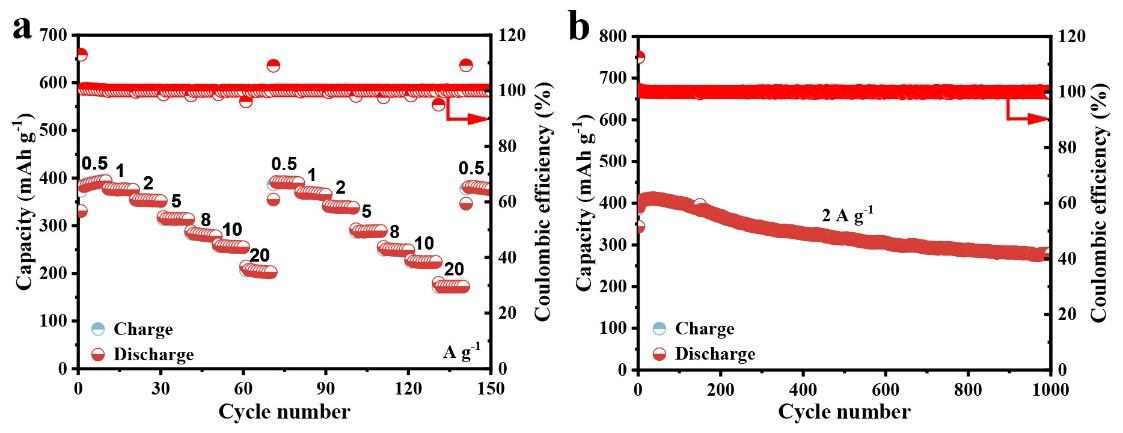


**Fig. S34** **a** Rate performances of pouch cell. **b** Cycling performance of pouch cell at a current density of 2 A g^-1^

**Fig. S35** Rate performance of pouch cell compared with literature [S14-S22]


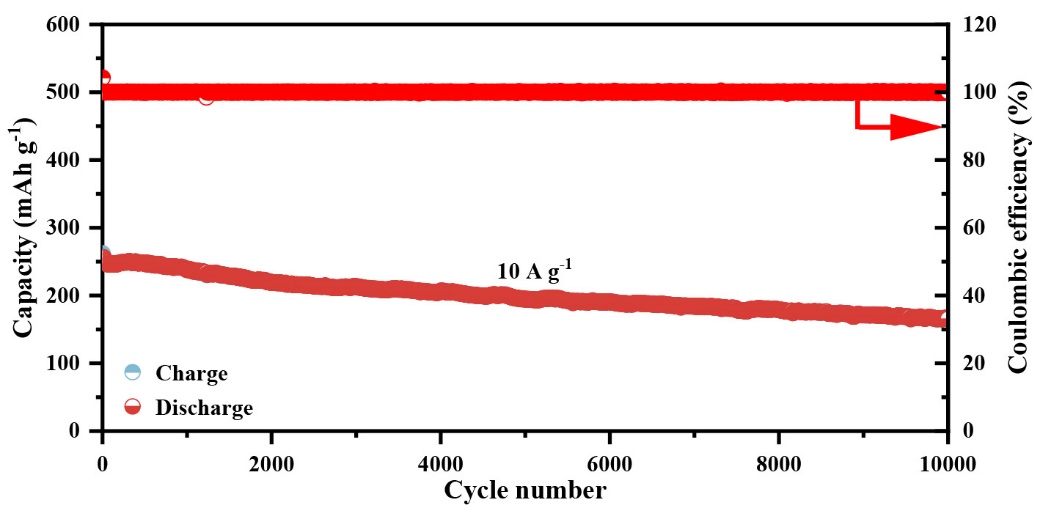


**Fig. S36** Long-cycling performance of pouch cell at a current density of 10 A g^-1^


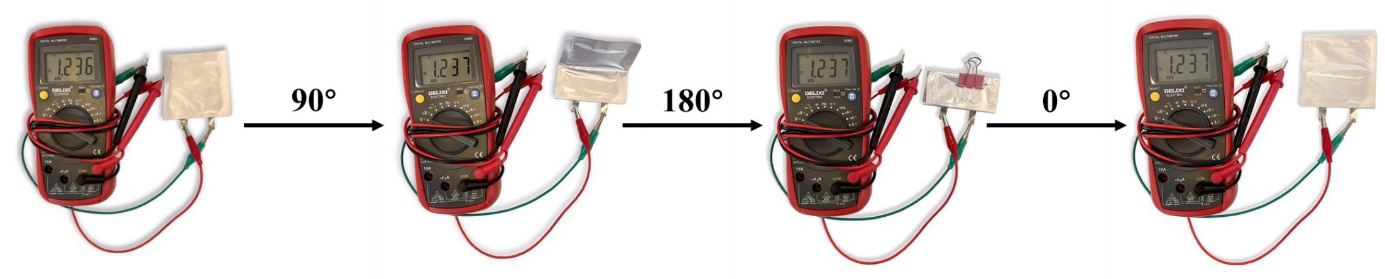


**Fig. S37** Open circuit voltage of the pouch cell under the 0°, 90°, and 180° bending states


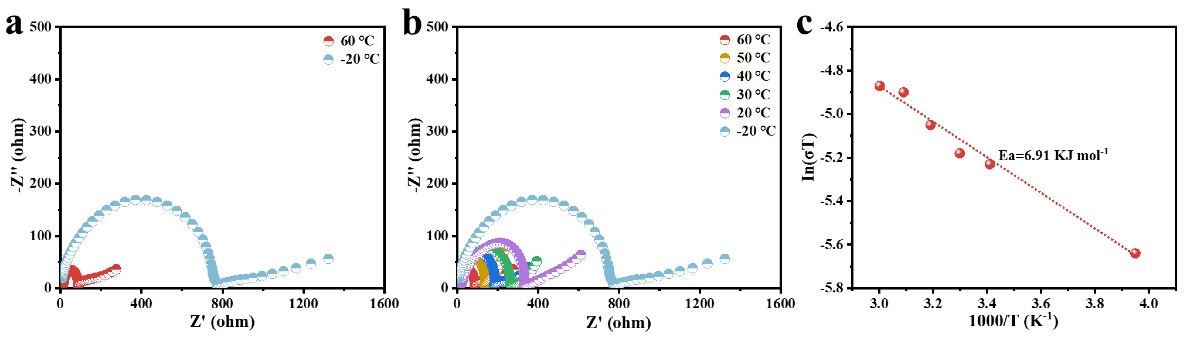


**Fig. S38** **a**, **b** EIS curves of pouch cell at different temperatures. **c** Activation energy calculated from the Arrhenius equation

**Table S1** OVs content calculated from XPS results

| Samples | NVO | KNVO | NVO-C_3_N_4_ | KNVO-C_3_N_4_ |
| --- | --- | --- | --- | --- |
| OVs  concentration | 32.46% | 34.58% | 35.10% | 36.86% |

**Table S2** Summary and comparison of rate performance of NVO, KNVO, NVO-C_3_N_4_, and KNVO-C_3_N_4_ electrodes

| Sample | Rate capacity/mAh g^-1^ (current density/A g^-1^) |
| --- | --- |
| NVO | 372.5 (0.5), 354.9 (1), 331.7 (2), 272.8 (5), 204.8 (8), 154.4 (10), 74.7 (20) |
| KNVO | 429.7 (0.5), 412.5 (1), 387.9 (2), 341.1 (5), 297.8 (8), 270.4 (10), 194.2 (20) |
| NVO-C_3_N_4_ | 403.3 (0.5), 386.7 (1), 364.4 (2), 322.1 (5), 287.6 (8), 254.3 (10), 172.6 (20) |
| **KNVO-C_3_N_4_** | **478.5 (0.5), 453.2 (1), 415.9 (2), 381.3 (5), 332.1 (8), 308.5 (10), 249.6 (20)** |

**Table S3** Comparison of electrochemical performance for different vanadium-based AZIBs

| Sample | Current density (A g^-1^) | Cycle number | Specific capacity (mA h g^-1^) | Rate capacity (current density) | References |
| --- | --- | --- | --- | --- | --- |
| **KNVO-C_3_N_4_** | **2** | **1000** | **348.5** | **249.6 (20)** | **This work** |
|  | **10** | **5000** | **214.2** |  |  |
|  | **20** | **10000** | **174.2** |  |  |
| NVO | 0.5 | 150 | 364.3 | 194.7 (20) | [S23] |
|  | 10 | 10000 | 219.9 |  |  |
| HNVO-Glu | 10 | 12000 | 202.1 | 230 (10) | [S24] |
| TMPA-VOH | 4 | 2000 | 290 | 294 (8) | [S25] |
| O_v_-ZVO | 2 | 2000 | 275 | 250 (3) | [S26] |
| CMVO | 1 | 200 | 402.5 | 100.1 (20) | [S27] |
|  | 15 | 5000 | - |  |  |
| NVO-Rb | 5 | 10000 | 148.3 | 152 (5) | [S28] |
| (1 Zn, 1Ch)-VOH | 4 | 2000 | 240 | 240 (5) | [S29] |
| MNVO | 0.5 | 300 | 296 | 140 (20) | [S30] |
|  | 10 | 7500 | - |  |  |
| O/V-defected NHVO | 15 | 8000 | 210 | 190 (15) | [S31] |
| KNVO | 5 | 3000 | 189 | 210 (5) | [S32] |
| RuVO | 10 | 5000 | 258 | 180.6 (20) | [S33] |
| Mo-V_2_O_3_-x@NC-3 | 1 | 500 | 386.8 | 190.9 (20) | [S34] |
|  | 20 | 10000 | 147.9 |  |  |
| HAVO-FeMo_6_-50 | 3 | 1000 | 140.4 | 262.1 (3) | [S35] |
| Mn_0.15_V_2_O5·nH_2_O | 1 | 450 | 265 | 150 (10) | [S36] |
|  | 10 | 8000 | 153 |  |  |
| CdHVO_y_@C | 1 | 100 | 326 | 198 (20) | [S37] |
|  | 20 | 3500 | 183 |  |  |
| WVO | 0.5 | 100 | 472.6 | 225.4 (10) | [S38] |
|  | 10 | 3000 | 219.6 |  |  |
| V_2_O_3_-SP2 | 10 | 2000 | 231.2 | 264.6 (10) | [S39] |
| KVOH | 5 | 3000 | 300 | 227 (10) | [S40] |
| PEDOT-NVO | 10 | 5000 | 160.6 | 163.4 (10) | [S41] |

**Supplementary References**

1. J. Hutter, M. Iannuzzi, F. Schiffmann, J. VandeVondele, cp2k: atomistic simulations of condensed matter systems. Wires Comput. Mol. Sci. **4**(1), 15–25 (2014). <https://doi.org/10.1002/wcms.1159>
2. G. Henkelman, B.P. Uberuaga, H. Jónsson, A climbing image nudged elastic band method for finding saddle points and minimum energy paths. J. Chem. Phys. **113**(22), 9901–9904 (2000). <https://doi.org/10.1063/1.1329672>
3. S. Goedecker, M. Teter, J. Hutter, Separable dual-space Gaussian pseudopotentials. Phys. Rev. B **54**(3), 1703–1710 (1996). <https://doi.org/10.1103/physrevb.54.1703>
4. A. Hjorth Larsen, J. Jørgen Mortensen, J. Blomqvist, I.E. Castelli, R. Christensen et al., The atomic simulation environment-a Python library for working with atoms. J. Phys. Condens. Matter **29**(27), 273002 (2017). <https://doi.org/10.1088/1361-648X/aa680e>
5. J.N. Canongia Lopes, J. Deschamps, A.A.H. Pádua, Modeling ionic liquids using a systematic all-atom force field. J. Phys. Chem. B **108**(6), 2038–2047 (2004). <https://doi.org/10.1021/jp0362133>
6. H.J.C. Berendsen, J.R. Grigera, T.P. Straatsma, The missing term in effective pair potentials. J. Phys. Chem. **91**(24), 6269–6271 (1987). <https://doi.org/10.1021/j100308a038>
7. A.K. Rappe, C.J. Casewit, K.S. Colwell, W.A. III Goddard, W.M. Skiff, UFF, a full periodic table force field for molecular mechanics and molecular dynamics simulations. J. Am. Chem. Soc. **114**(25), 10024–10035 (1992). <https://doi.org/10.1021/ja00051a040>
8. S. Plimpton, Fast parallel algorithms for short-range molecular dynamics. J. Comput. Phys. **117**(1), 1–19 (1995). <https://doi.org/10.1006/jcph.1995.1039>
9. Y. Shi, R. Wang, S. Bi, M. Yang, L. Liu et al., An anti-freezing hydrogel electrolyte for flexible zinc-ion batteries operating at −70 ℃. Adv. Funct. Mater. **33**(24), 2214546 (2023). <https://doi.org/10.1002/adfm.202214546>
10. T. Sun, S. Zheng, H. Du, Z. Tao, Synergistic effect of cation and anion for low-temperature aqueous zinc-ion battery. Nano-Micro Lett. **13**(1), 204 (2021). <https://doi.org/10.1007/s40820-021-00733-0>
11. S. Cao, J. Low, J. Yu, M. Jaroniec, Polymeric photocatalysts based on graphitic carbon nitride. Adv. Mater. **27**(13), 2150–2176 (2015). <https://doi.org/10.1002/adma.201500033>
12. J. Yang, S. Xing, J. Zhou, Y. Cheng, L. Shi et al., The controlled construction of a ternary hybrid of monodisperse Ni3S4 nanorods/graphitic C_3_N_4_ nanosheets/nitrogen-doped graphene in van der Waals heterojunctions as a highly efficient electrocatalyst for overall water splitting and a promising anode material for sodium-ion batteries. J. Mater. Chem. A **7**(8), 3714–3728 (2019). <https://doi.org/10.1039/C8TA07253A>
13. S. Wang, Z. Yuan, X. Zhang, S. Bi, Z. Zhou et al., Non-metal ion co-insertion chemistry in aqueous Zn/MnO(2) batteries. Angew. Chem. Int. Ed. **60**(13), 7056–7060 (2021). <https://doi.org/10.1002/anie.202017098>
14. S. Zhao, S. Wang, J. Guo, L. Li, C. Li et al., Sodium-ion and polyaniline co-intercalation into ammonium vanadate nanoarrays induced enlarged interlayer spacing as high-capacity and stable cathodes for flexible aqueous zinc-ion batteries. Adv. Funct. Mater. **33**(48), 2305700 (2023). <https://doi.org/10.1002/adfm.202305700>
15. R. Wang, H. Dai, T. Zhang, J. Zhou, L. Yin et al., Heterostructure design of amorphous vanadium Oxides@Carbon/graphene nanoplates boosts improved capacity, cycling stability and high rate performance for Zn^2+^ storage. Adv. Funct. Mater. **35**(19), 2421857 (2025). <https://doi.org/10.1002/adfm.202421857>
16. C. Liu, W. Xu, C. Mei, M.-C. Li, X. Xu et al., Highly stable H_2_V_3_O_8_/Mxene cathode for Zn-ion batteries with superior rate performance and long lifespan. Chem. Eng. J. **405**, 126737 (2021). <https://doi.org/10.1016/j.cej.2020.126737>
17. C. Chen, T. Wang, X. Zhao, A. Wu, S. Li et al., Customizing hydrophilic terminations for V2CTx MXene toward superior hybrid-ion storage in aqueous zinc batteries. Adv. Funct. Mater. **34**(9), 2308508 (2024). <https://doi.org/10.1002/adfm.202308508>
18. S. Kong, Y. Li, X. Zhang, Z. Xu, X. Wang et al., Anchoring polar organic molecules in defective ammonium vanadate for high-performance flexible aqueous zinc-ion battery. Small **19**(52), 2304462 (2023). <https://doi.org/10.1002/smll.202304462>
19. W. Qiu, Y. Tian, S. Lin, A. Lei, Z. Geng et al., Water molecules and oxygen-vacancy modulation of vanadium pentoxide with fast kinetics toward ultrahigh power density and durable flexible all-solid-state zinc ion battery. J. Energy Chem. **85**, 581–591 (2023). <https://doi.org/10.1016/j.jechem.2023.06.042>
20. T. Wang, S. Li, X. Weng, L. Gao, Y. Yan et al., Ultrafast 3D hybrid-ion transport in porous V_2_O_5_ cathodes for superior-rate rechargeable aqueous zinc batteries. Adv. Energy Mater. **13**(18), 2204358 (2023). <https://doi.org/10.1002/aenm.202204358>
21. B. Li, K. Yang, J. Ma, P. Shi, L. Chen et al., Multicomponent copper-zinc alloy layer enabling ultra-stable zinc metal anode of aqueous Zn-ion battery. Angew. Chem. Int. Ed. **61**(47), e202212587 (2022). <https://doi.org/10.1002/anie.202212587>
22. R. Puttaswamy, H. Lee, H.-W. Bae, D.Y. Kim, D. Kim, Ethylene glycol-choline chloride based hydrated deep eutectic electrolytes enabled high-performance zinc-ion battery. Small **20**(35), 2400692 (2024). <https://doi.org/10.1002/smll.202400692>
23. Y. Xu, G. Fan, P.X. Sun, Y. Guo, Y. Wang et al., Carbon nitride pillared vanadate *via* chemical pre-intercalation towards high-performance aqueous zinc-ion batteries. Angew. Chem. Int. Ed. **62**(26), e202303529 (2023). <https://doi.org/10.1002/anie.202303529>
24. P. Zhang, Y. Gong, S. Fan, Z. Luo, J. Hu et al., Glutamic acid induced proton substitution of sodium vanadate cathode promotes high performance in aqueous zinc-ion batteries. Adv. Energy Mater. **14**(30), 2401493 (2024). <https://doi.org/10.1002/aenm.202401493>
25. X. Jia, C. Liu, Z. Wang, D. Huang, G. Cao, Weakly polarized organic cation-modified hydrated vanadium oxides for high-energy efficiency aqueous zinc-ion batteries. Nano-Micro Lett. **16**(1), 129 (2024). <https://doi.org/10.1007/s40820-024-01339-y>
26. J.-J. Ye, P.-H. Li, H.-R. Zhang, Z.-Y. Song, T. Fan et al., Manipulating oxygen vacancies to spur ion kinetics in V_2_O_5_ structures for superior aqueous zinc-ion batteries. Adv. Funct. Mater. **33**(46), 2305659 (2023). <https://doi.org/10.1002/adfm.202305659>
27. A. Wang, D.-H. Liu, L. Yang, F. Xu, D. Luo et al., Building stabilized Cu_0.17_Mn_0.03_V_2_O_5_− ·2.16H_2_O cathode enables an outstanding room-/ low-temperature aqueous Zn-ion batteries. Carbon Energy **6**(8), e512 (2024). <https://doi.org/10.1002/cey2.512>
28. K. Wang, S. Li, X. Chen, J. Shen, H. Zhao et al., Trifunctional Rb(+)-intercalation enhancing the electrochemical cyclability of ammonium vanadate cathode for aqueous zinc ion batteries. ACS Nano **18**(9), 7311–7323 (2024). <https://doi.org/10.1021/acsnano.4c00803>
29. Q. Zong, Y. Zhuang, C. Liu, Q. Kang, Y. Wu et al., Dual effects of metal and organic ions co-intercalation boosting the kinetics and stability of hydrated vanadate cathodes for aqueous zinc-ion batteries. Adv. Energy Mater. **13**(31), 2301480 (2023). <https://doi.org/10.1002/aenm.202301480>
30. X. Wang, Y. Wang, A. Naveed, G. Li, H. Zhang et al., Magnesium ion doping and micro-structural engineering assist NH_4_V_4_O_10_ as a high-performance aqueous zinc ion battery cathode. Adv. Funct. Mater. **33**(48), 2306205 (2023). <https://doi.org/10.1002/adfm.202306205>
31. S. Li, X. Xu, W. Chen, J. Zhao, K. Wang et al., Synergetic impact of oxygen and vanadium defects endows NH_4_V_4_O_10_ cathode with superior performances for aqueous zinc-ion battery. Energy Storage Mater. **65**, 103108 (2024). <https://doi.org/10.1016/j.ensm.2023.103108>
32. Q. Zong, Q. Wang, C. Liu, D. Tao, J. Wang et al., Potassium ammonium vanadate with rich oxygen vacancies for fast and highly stable Zn-ion storage. ACS Nano **16**(3), 4588–4598 (2022). <https://doi.org/10.1021/acsnano.1c11169>
33. Z. Chen, H. Liu, S. Fan, Q. Zhang, C. Yuan et al., Inhibition of vanadium cathode dissolution in zinc-ion batteries on thermodynamics and kinetics by guest pre-intercalation. Adv. Energy Mater. **14**(25), 2400977 (2024). <https://doi.org/10.1002/aenm.202400977>
34. D. Chen, M. Yang, Y. Ming, W. Cai, S. Shi et al., Synergetic effect of Mo-doped and oxygen vacancies endows vanadium oxide with high-rate and long-life for aqueous zinc ion battery. Small **20**(48), 2405168 (2024). <https://doi.org/10.1002/smll.202405168>
35. Y. Zhang, Q. Li, W. Feng, H. Yue, S. Gao et al., Valence engineering *via* polyoxometalate-induced on vanadium centers for efficient aqueous zinc-ion batteries. Angew. Chem. Int. Ed. **64**(19), e202501728 (2025). <https://doi.org/10.1002/anie.202501728>
36. H. Geng, M. Cheng, B. Wang, Y. Yang, Y. Zhang et al., Electronic structure regulation of layered vanadium oxide *via* interlayer doping strategy toward superior high-rate and low-temperature zinc-ion batteries. Adv. Funct. Mater. **30**(6), 1907684 (2020). <https://doi.org/10.1002/adfm.201907684>
37. T. Chen, Q. Wu, L. Lang, Z. Chen, G. Luo et al., Modified structure of vanadium oxide *via* cadmium doping and *in situ* activation for high-performance aqueous zinc ion storage. Chem. Eng. J. **499**, 156295 (2024). <https://doi.org/10.1016/j.cej.2024.156295>
38. Y. Li, X. Li, M. Xie, X. Liao, X. He et al., Morphology regulation and vacancy engineering for vanadium oxide cathodes *via* tungsten doping towards advanced zinc-ion batteries. J. Colloid Interface Sci. **696**, 137888 (2025). <https://doi.org/10.1016/j.jcis.2025.137888>
39. D. Li, Z. Ye, H. Ding, J. Li, H. Huang et al., Boosting proton intercalation *via* sulfur anion doping in V_2_O_3_ cathode materials towards high capacity and rate performance of aqueous zinc ion batteries. Energy Storage Mater. **71**, 103635 (2024). <https://doi.org/10.1016/j.ensm.2024.103635>
40. M. Tian, C. Liu, J. Zheng, X. Jia, E.P. Jahrman et al., Structural engineering of hydrated vanadium oxide cathode by K+ incorporation for high-capacity and long-cycling aqueous zinc ion batteries. Energy Storage Mater. **29**, 9–16 (2020). <https://doi.org/10.1016/j.ensm.2020.03.024>
41. D. Bin, W. Huo, Y. Yuan, J. Huang, Y. Liu et al., Organic-inorganic-induced polymer intercalation into layered composites for aqueous zinc-ion battery. Chem **6**(4), 968–984 (2020). <https://doi.org/10.1016/j.chempr.2020.02.001>
